# A systematic strategy for estimating *hERG* block potency and its implications in a new cardiac safety paradigm

**DOI:** 10.1016/j.taap.2020.114961

**Published:** 2020-05-01

**Authors:** Bradley J. Ridder, Derek J. Leishman, Matthew Bridgland-Taylor, Mohammadreza Samieegohar, Xiaomei Han, Wendy W. Wu, Aaron Randolph, Phu Tran, Jiansong Sheng, Timm Danker, Anders Lindqvist, Daniel Konrad, Simon Hebeisen, Liudmila Polonchuk, Evgenia Gissinger, Muthukrishnan Renganathan, Bryan Koci, Haiyang Wei, Jingsong Fan, Paul Levesque, Jae Kwagh, John Imredy, Jin Zhai, Marc Rogers, Edward Humphries, Robert Kirby, Sonja Stoelzle-Feix, Nina Brinkwirth, Maria Giustina Rotordam, Nadine Becker, Søren Friis, Markus Rapedius, Tom A. Goetze, Tim Strassmaier, George Okeyo, James Kramer, Yuri Kuryshev, Caiyun Wu, Herbert Himmel, Gary R. Mirams, David G. Strauss, Rémi Bardenet, Zhihua Li

**Affiliations:** a Division of Applied Regulatory Science, Office of Clinical Pharmacology, Office of Translational Sciences, Center for Drug Evaluation and Research, Food and Drug Administration, 10903 New Hampshire Ave, Silver Spring, MD 20993, USA; b Department of Toxicology and Pathology, Eli Lilly and Company, Indianapolis, IN, USA; c Clinical Pharmacology & Safety Sciences, R&D, AstraZeneca, Cambridge, United Kingdom; d CiPA LAB, 900 Clopper Rd, Suite 130, Gaithersburg, MD 20878, USA; e NMI-TT GmbH, Markwiesenstr. 55, 72770 Reutlingen, Germany; f Sophion Bioscience A/S, Baltorpvej 154, 2750 Ballerup, Denmark; g B'SYS GmbH, The Ion Channel Company, Benkenstrasse 254, CH-4108, Witterswil, Switzerland; h F. Hoffmann-La Roche AG, F. Hoffmann-La Roche Ltd Bldg. 73/R. 103b Grenzacherstrasse, 124, CH-4070 Basel, Switzerland; i Eurofins Scientific, Eurofins Discovery, 6 Research Park Drive, St. Charles, MO 63304, USA; j Bristol-Myers Squibb Company, Discovery Toxicology, Bristol-Myers Squibb, 3551 Lawrenceville, Princeton Rd, Lawrence Township, NJ 08648, USA; k Merck & Co., Inc, Kenilworth, NJ, USA; l Metrion Biosciences Limited, Riverside 3, Suite 1, Granta Park, Great Abington, Cambridge CB21, 6AD, United Kingdom; m Nanion Technologies Munich, Ganghoferstrasse 70A, 80339 Munich, Germany; n Nanion Technologies, USA, 1 Naylon Place, Suite C, Livingston, NJ 07039, USA; o Charles River Laboratories, 14656 Neo Parkway, Cleveland, OH 44128, USA; p Bayer AG, RD-TS-TOX-SP-SPL1, Aprather Weg 18a, 42096 Wuppertal, Germany; q Centre for Mathematical Medicine & Biology, School of Mathematical Sciences, University of Nottingham, Nottingham, United Kingdom; r Université de Lille, CNRS, Centrale Lille, UMR 9189 - CRIStAL, Villeneuve d'Ascq, France

## Abstract

**Introduction:**

*hERG* block potency is widely used to calculate a drug's safety margin against its torsadogenic potential. Previous studies are confounded by use of different patch clamp electrophysiology protocols and a lack of statistical quantification of experimental variability. Since the new cardiac safety paradigm being discussed by the International Council for Harmonisation promotes a tighter integration of nonclinical and clinical data for torsadogenic risk assessment, a more systematic approach to estimate the *hERG* block potency and safety margin is needed.

**Methods:**

A cross-industry study was performed to collect *hERG* data on 28 drugs with known torsadogenic risk using a standardized experimental protocol. A Bayesian hierarchical modeling (BHM) approach was used to assess the *hERG* block potency of these drugs by quantifying both the inter-site and intra-site variability. A modeling and simulation study was also done to evaluate protocol-dependent changes in *hERG* potency estimates.

**Results:**

A systematic approach to estimate *hERG* block potency is established. The impact of choosing a safety margin threshold on torsadogenic risk evaluation is explored based on the posterior distributions of *hERG* potency estimated by this method. The modeling and simulation results suggest any potency estimate is specific to the protocol used.

**Discussion:**

This methodology can estimate *hERG* block potency specific to a given voltage protocol. The relationship between safety margin thresholds and torsadogenic risk predictivity suggests the threshold should be tailored to each specific context of use, and safety margin evaluation may need to be integrated with other information to form a more comprehensive risk assessment.

## Highlights

• hERG potency/safety margin is a widely used nonclinical cardiac safety strategy.
• A new regulatory paradigm promotes the integration of nonclinical and clinical data.
• Lack of uncertainty quantification hindered using hERG potency in the new paradigm.
• A systematic method was established to address this limitation.
• Analysis supports using different safety margin thresholds in different context.

## 1 Introduction

The cardiac action potential is regulated by the electrical current flows of ions across cardiomyocyte membranes. Many drugs can bind to ion channels, block ionic flow and disrupt the regulation of the action potential, leading to a drug-induced arrhythmia, or “proarrhythmia” (Friedman and Stevenson, 1998). A particularly dangerous type of proarrhythmia is known as “Torsade de Pointes,” or TdP, which is a rare ventricular tachycardia with a potential to cause sudden cardiac death (Roden, 2008). The ion channel of greatest interest to the identification of TdP risk is the Kv11.1 potassium channel, which is encoded by *hERG* (human ether-a-go-go related gene) and carries the rapidly activating delayed rectifier potassium current (*I*_*Kr*_) (Vandenberg et al., 2012). Block of the *hERG* channel results in a reduction of *I*_*Kr*_ and repolarization reserve (Roden, 1998), and in turn may lead to QT prolongation and TdP (Nachimuthu et al., 2012). Although *I*_*Kr*_ is one of the most prominent repolarizing currents, other cardiac currents also contribute to repolarization (Roden, 1998). Based on this more comprehensive understanding of cardiac electrophysiology and cellular mechanisms of TdP, the Comprehensive in vitro Proarrhythmia Assay (CiPA) was proposed to integrate multi-ion channel pharmacology measured in vitro into experimentally-parameterized in silico models to assess TdP risk (Sager et al., 2014). The progress made by the CiPA Initiative and other similar projects worldwide have led to the formation of an International Council for Harmonisation (ICH) Implementation Working Group to develop Questions & Answers (Q&As) for ICH S7B (nonclinical) and E14 (clinical) guidelines (Questions and Answers, 2018).

This upcoming new international cardiac safety paradigm may facilitate the use of nonclinical data as part of an integrated risk assessment strategy to inform clinical decision making. Two types of nonclinical approaches have been used in cardiac safety assessment. One type focuses solely on quantifying block of the hERG channel, as this is the most common ionic basis for TdP (Redfern et al., 2003). The other uses a more comprehensive platform (such as in silico models with multi-ion channel in vitro data (Kramer et al., 2013; Abbasi et al., 2017), induced pluripotent stem cell (iPS)-derived cardiomyocytes (Ando et al., 2017), or in vivo/ex vivo systems (Champeroux et al., 2005)) to quantify the pharmacological effects on the cardiac system. While the latter can be regarded as proarrhythmia risk prediction models and typically produce a metric (such as a numerical score or qualitative classification) to predict the risk, the former usually try to identify a “safety margin” threshold, where safety margin is defined as the ratio of the half inhibitory concentration,*IC*_50_, for hERG inhibition to the maximum free therapeutic concentration, *C*_*max*_^*free*^, of the drug. The assumption is that above the safety margin threshold, the compound is not likely to induce TdP (Redfern et al., 2003).

There have been previous attempts to relate *hERG* safety margin to TdP risk (Wallis, 2010). However, several confounding factors make it difficult to interpret these past findings. First, these investigations (Redfern et al., 2003; De Bruin et al., 2005; Gintant, 2011; Webster et al., 2002) pooled together drug potency data from vastly different experimental conditions (voltage protocol, temperature, native vs. heterologous systems, etc.). As the results of in vitro ion channel patch clamp assays are sensitive to these conditions (Kirsch et al., 2004; Lee et al., 2019), the inconsistent data used in these studies renders their proposed safety margin thresholds of dubious validity. Second, some studies used clinical QTc prolongation as the endpoint (Gintant, 2011; Webster et al., 2002). Since the real concern is TdP, the use of a surrogate marker limits the use of the proposed safety margin threshold. Third, no uncertainty quantification was done in these studies, and all results are point estimates. The use of a single point estimate may be acceptable for early stage drug screening studies. For late-stage regulatory risk assessment, however, the uncertainty in the data must be accounted for. Lastly, all these studies defined safety margin using hERG *IC*_50_ as the drug potency parameter. This prevented the use of such a strategy on drugs for which an accurate measurement of *IC*_50_ is not possible, for instance due to solubility issues.

Under the CiPA Initiative, a systematic strategy was developed to address the above issues. A standardized voltage protocol was used in measurements of 28 CiPA drugs with known clinical TdP risk at several globally-distributed facilities (“sites”). All sites used high throughput (HTS) automated patch-clamp systems, though not necessarily the same type of devices. The data were generated in a blinded fashion by each site, and then collected for centralized de-blinding and data analysis. A rigorous statistical method was applied to account for inter- and intra- site variability. A method was also developed to calculate the block potency using not only *IC*_50_, but also lower inhibitory concentrations (such as *IC*_10_, *IC*_20_) to accommodate for those drugs with solubility issue. Based on the estimated posterior distributions of *hERG* potency of the 28 drugs, the relationship between choosing a specific safety margin threshold and making an error in TdP risk classification (false positive and negative rates) is explored. Finally, a modeling and simulation study was used to highlight the fact that any *hERG* block potency estimation depends on the experimental temperature and the particular voltage protocol used.

## 2 Methods

### 2.1 Pharmacology and electrophysiology

The CiPA Initiative organized a panel of facilities with HTS systems to participate in a multi-site study coordinated by the Health and Environmental Sciences Institute (HESI). The study was conducted in two phases (phase 1 for 12 CiPA training drugs and phase 2 for 16 CiPA validation drugs) and not all sites participated in both phases. The 28 drugs were categorized into High, Intermediate, or Low TdP risk classes by a dedicated CiPA team (Colatsky et al., 2016). While training and validation data sets were needed for the development of CiPA in silico models (Chang et al., 2017; Dutta et al., 2017; Li et al., 2017; Li et al., 2018), such a division is not needed here. Therefore, the two data sets were unified in this study. Dose-response data for several other ion channels and physiological temperature was also collected as part of the study, but only the ambient temperature *hERG* data was analyzed here. The identities of the participating sites were masked by numerical indicators. A centralized procedure was taken where all 28 drugs were purchased and prepared into stock solutions by a single laboratory outside of the participating sites. The identities and concentrations of the stock solutions were masked. Each site received a blinded aliquot of the stock solutions. Instructions were provided for making serial dilutions from each stock solution. The sites performed their *hERG* assays without knowing the identities or concentrations of the compounds. The unblinded drug names and concentrations, along with the measured block percentage for each cell, are available along with this publication at https://github.com/FDA/CiPA/tree/Systematic_Strategy_hERG_Block_2020.

The sources of the 28 CiPA drugs are as follows. For Phase 1, all drugs were purchased from Millipore Sigma (St. Louis, MO), formulated in 100% DMSO, blinded and shipped frozen to collaborator testing sites. All stocks were prepared at 1000× concentration to be tested, to limit final DMSO exposure upon preparation of serial dilutions to 0.1% in each assay. For Phase 2, blinded drug powder with instruction for formulation of stock solutions was sent to the sites by the Chemotherapeutic Agents Repository of the National Cancer Institute and stored at −20 °C until the day of testing.

The CiPA step-ramp protocol was used as the standard protocol across sites. It involves a pulse pattern, repeated every 5 s, consisting of a depolarization to 40 mV amplitude for a 500 ms duration, followed by a ramp (1.2 V/s) to −80 mV for 100 ms. The holding potential is −80 mV. Peak tail current is measured during the ramp. Site-specific parameters, such as cell lines, buffers, HTS platforms, can be found in Supplementary Materials. The details of the action potential waveform protocol can be found in a previous publication (Sheng et al., 2017).

### 2.2 Bayesian hierarchical model to analyze multi-site data

We used a Bayesian hierarchical model (BHM) similar to that used by Johnstone et al. for analysis of ion channel dose-response data (Johnstone et al., 2016). The distribution of a logarithmic transformation of the *IC*_50_ for the *j*^*th*^ site (denoted by *pIC*_50_^*j*^) across all sites was assumed to follow a logistic distribution. Similarly, the site-specific Hill coefficients (*h*_*j*_) across all sites were assumed to follow a log-logistic distribution. The hyperparameters controlling these distributions (inter-site variability), along with site-specific probability distributions of *IC*_50_ (and Hill coefficients) within each site (intra-site variability), are estimated through a Markov Chain Monte Carlo (MCMC) process using the Metropolis-Hastings algorithm. In-house developed R scripts using the FME package (https://cran.r-project.org/web/packages/FME/index.html) were run on the FDA's High Performance Computing cluster to execute the algorithms. A discussion of the prior information, bounds on the parameters, and implementation details can be found in the Supplementary Methods.

### 2.3 Modeling and simulation method for predicting the effect different voltage protocols have on *hERG* block potency estimates

The *hERG* dynamic model previously developed for CiPA (Li et al., 2017) was used to simulate drug effects on the *hERG* channel using various input voltage protocols. The drug-specific kinetic parameters were estimated by fitting to time-dependent fractional block data from a modified Milnes protocol (Milnes et al., 2010). A quality criterium was applied to select only those cells with less than 20% background current, where the background current was measured by applying 0.5 μM E-4031 to the cell at the end of each experiment. A bootstrapping procedure was used to quantify the uncertainty in the drug-specific kinetic binding parameters. This generated 2000 sets of kinetic binding parameters for each drug, which provides a numerical approximation of the true joint distribution (Chang et al., 2017). For each drug, the 2000 sets of parameters were used to perform 2000 simulations for a particular voltage protocol across 10 concentrations. The concentrations were chosen to span a wide range of block for each drug. For each drug and voltage protocol, this gave a simulated dose-response dataset of 2000 predicted block values for each of the ten different concentrations. The joint probability distribution of *IC*_50_s and Hill coefficients for each drug was found by applying Markov-chain Monte Carlo to the simulated dose-response data. The likelihood function assumed the mean block is normally distributed with the mean equal to the block predicted by the Hill equation, and error about the mean described by a drug-specific variance term. Hill coefficient was bounded between 0.5 and 2.0, which was the range obtained by previous investigators after examination a large amount of HTS dose-response data (see (Elkins et al., 2013) and Supplementary Methods for details). The raw patch clamp data obtained from the Milnes protocol, code, fitted *hERG* kinetic drug binding parameters, and simulated dose-response curves can be all found at https://github.com/FDA/CiPA/tree/ Systematic_Strategy_hERG_Block_2020. We note to the reader that the *hERG* model uses an *E*_max_ model to assume a saturating maximum drug effect (Li et al., 2017). As a consequence, the Hill equation used to fit the simulated dose-response data was slightly altered to account for a maximum block effect (*B*_max_). This is similar to a modified Hill equation used to introduce an *I*_max_ effect. Details can be found in the Supplementary Methods and in reference (Mistry, 2018). Of note the *B*_max_ term was only used to estimate *hERG* potency from simulated dose-response dataset. It was not used when estimating *hERG* potency from real experimental data through BHM.

## 3 Results

### 3.1 Multi-site *hERG* data from HTS using the CiPA step-ramp protocol

**Fig. 1:**
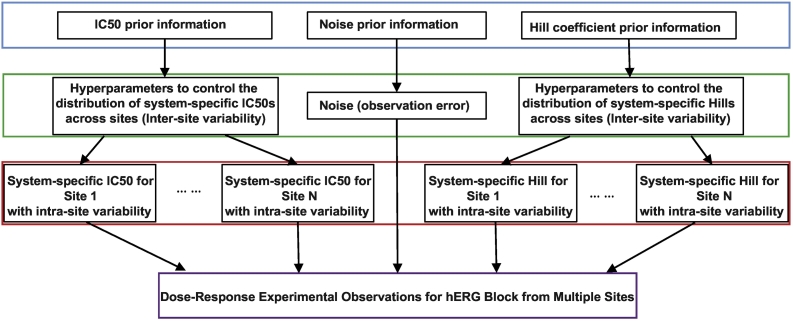
The Bayesian Hierarchical Modeling (BHM) structure to quantitate both inter- and intra- site variability in multi-site *hERG* assay data. A diagram depicting the structure of the BHM model to infer distributions of statistical parameters that give rise to the observed experimental data. The blue box at the top is the “prior information,” which is prior knowledge or assumptions we have about the experimental systems. The green box corresponds to the distribution of hyperparameters that control the system-specific *IC*_50_s and Hill coefficients across sites (inter-site variability). Similar to Johnstone et al. (Johnstone et al., 2016), we assume *IC*_50_s and Hill coefficients for the same drug follow two distinct distributions across sites and hence are governed by two independent sets of hyperparameters (see Supplementary Methods), although it is possible that there is some correlation between *IC*_50_s and Hill coefficients for the same drug across sites. The red box corresponds to the distribution of site/system-specific parameters (*IC*_50_s and Hill coefficients) within each site (intra-site variability). Note that each site has its own distribution of *IC*_50_s and Hill coefficients. Site 1 and Site N (the last site) were shown with other sites being represented by ellipsis. The purple box at the bottom is the set of all experimental observations provided by all sides. Of note, the prior information for *IC*_50_ and Hill coefficient was deduced by following the approach of Johnstone et al. (Johnstone et al., 2016) using HTS screening data with a large number of repeats (Elkins et al., 2013) (see Supplementary Methods for details). In addition, for Hill coefficients we set a boundary between 0.5 and 2.0, after examining HTS screening data with large numbers of repeats (see Supplementary Methods for details). For the prior information of measurement error or system noise, we used a uniform distribution for all sites, although in theory prior information about system noise can be obtained for each site and used to further constrain the parameters. One of the hyperparameters in the green box (the location parameter *μ*, see Supplementary Methods) corresponds to the mean of the *IC*_50_ distribution across sites. The probability distribution of *μ* reflects our uncertainty in estimating the mean hERG block potency across sites, and will be used as each drug's IC50 distribution to calculate the safety margin distribution across sites. (For interpretation of the references to colour in this figure legend, the reader is referred to the web version of this article.)

An initial examination of the multi-site *hERG* data using the same standard voltage protocol suggests there are significant intra-site and inter-site variabilities (Fig. S1 in Supplementary Materials). Theoretically, intra-site variability may be mainly attributed to the inherent randomness of the measurement (random measurement error from the system and random variation of electrophysiological properties from cell to cell) while inter-site variability may largely stem from systematic differences between sites (different platforms, cell lines, internal quality control, etc.). To account for both types of variability, we adopted a Bayesian hierarchical modeling (BHM) approach (Johnstone et al., 2016). The general idea behind BHM is to assume that, for any drug, each site has its own specific *hERG IC*_50_ and Hill coefficients, which are determined by the specific configuration of the experimental system at that particular site. The variation of these system-specific (or site-specific) *IC*_50_s and Hill coefficients across sites can be modeled as a “higher level” distribution to quantify inter-site variability. Within each individual site, the system-specific *IC*_50_ and Hill coefficient distributions for each drug determine a “lower level” uncertainty in dose-response relationships that characterize intra-site variability. The hyperparameters that describe inter-site variability and the site-specific parameters that describe intra-site variability can be estimated from the multi-site data, as shown as a diagram in Fig. 1. By applying this BHM modeling strategy to the multi-site data, we derived joint distributions of the site-specific parameters and hyperparameters for each of the 28 drugs. One of the hyperparameters *μ* (see Supplementary Methods), which corresponds to the mean of the estimated *IC_50_* distribution across sites for each drug, is shown in Table 1.

**Table 1:** *IC*_50_ and *IC*_20_ values for the CiPA drugs after incorporating inter- and intra- site uncertainty using a Bayesian Hierarchical Model (BHM).

Drug	Risk	*IC*_20_, BHM [nM]	*IC*_50_, BHM [nM]
vandetanib	high	73 (58–91)	394 (330–472)
sotalol	high	5.4E4 (3.8E4–7.8E4)	2.9E5 (2.1E5 - 4E5)
quinidine	high	223 (175–280)	971 (791–1.2E3)
ibutilide	high	3.9 (2.4–6.5)	11 (6.5–17)
dofetilide	high	17 (10–28)	75 (50–117)
disopyramide	high	991 (453–2.3E3)	4.7E3 (2.6E3–9.3E3)
bepridil	high	48 (39–57)	144 (120–172)
azimilide	high	86 (67–109)	380 (303–476)
terfenadine	inter.	43 (33–55)	129 (103–159)
risperidone	inter.	109 (70–167)	451 (308–646)
pimozide	inter.	4.6 (2.7–7.7)	19 (13–29)
ondansetron	inter.	288 (225–378)	1.2E3 (930–1.6E3)
droperidol	inter.	34 (26–44)	118 (96–148)
domperidone	inter.	21 (14–32)	74 (52–106)
clozapine	inter.	371 (248–570)	1.5E3 (952–2.3E3)
clarithromycin	inter.	2.4E4 (1E4–5.3E4)	1.5E5 (7.2E4–3.1E5)
cisapride	inter.	13 (10–17)	56 (44–72)
chlorpromazine	inter.	244 (160–395)	650 (441–1.1E3)
astemizole	inter.	4.6 (2.5–8)	19 (11−32)
verapamil	low	129 (99–166)	452 (343–599)
tamoxifen	low	545 (410–722)	1.7E3 (1.3E3–2.3E3)
ranolazine	low	1.9E3 (1.5E3–2.3E3)	8.3E3 (6.6E3–1E4)
nitrendipine	low	3.7E3 (2.3E3–6.1E3)	2E4 (1.3E4–2.9E4)
nifedipine	low	1.6E4 (1E4–2.5E4)	7.1E4 (4.6E4–1.1E5)
mexiletine	low	1.1E4 (9.1E3–1.2E4)	5.3E4 (4.7E4–6.1E4)
metoprolol	low	2.5E4 (1.6E4–3.7E4)	1.1E5 (7.5E4–1.7E5)
loratadine	low	254 (147–473)	1.3E3 (825–2.3E3)
diltiazem	low	2.1E3 (1.6E3–2.6E3)	9.9E3 (7.9E3–1.2E4)

The *hERG* assay data for 28 CiPA drugs across multiple sites using high throughput automated patch clamp systems were collected and subjected to a BHM as depicted in Fig. 1. The lower boundary, median, and upper boundary of the 95% credible intervals (CI) of the mean *IC*_50_s and *IC*_20_s across sites for all drugs are shown. Units are in nM.

Drugs with poor solubility or weakly interact with the *hERG* channel often cannot attain close to 50% block in experimental practice. This makes *IC*_50_ for such drugs difficult to estimate. To remedy this problem, *IC*_20_ has been proposed as a substitute for *IC*_50_ to quantify *hERG* block potency (Redfern et al., 2003; Wallis, 2010). Our proposed method allows the calculation of low inhibitory concentrations (*IC*_10_, *IC*_20_, etc.) after taking into consideration the experimental variability (Table 1 and Supplementary Methods).

**Fig. 2:**
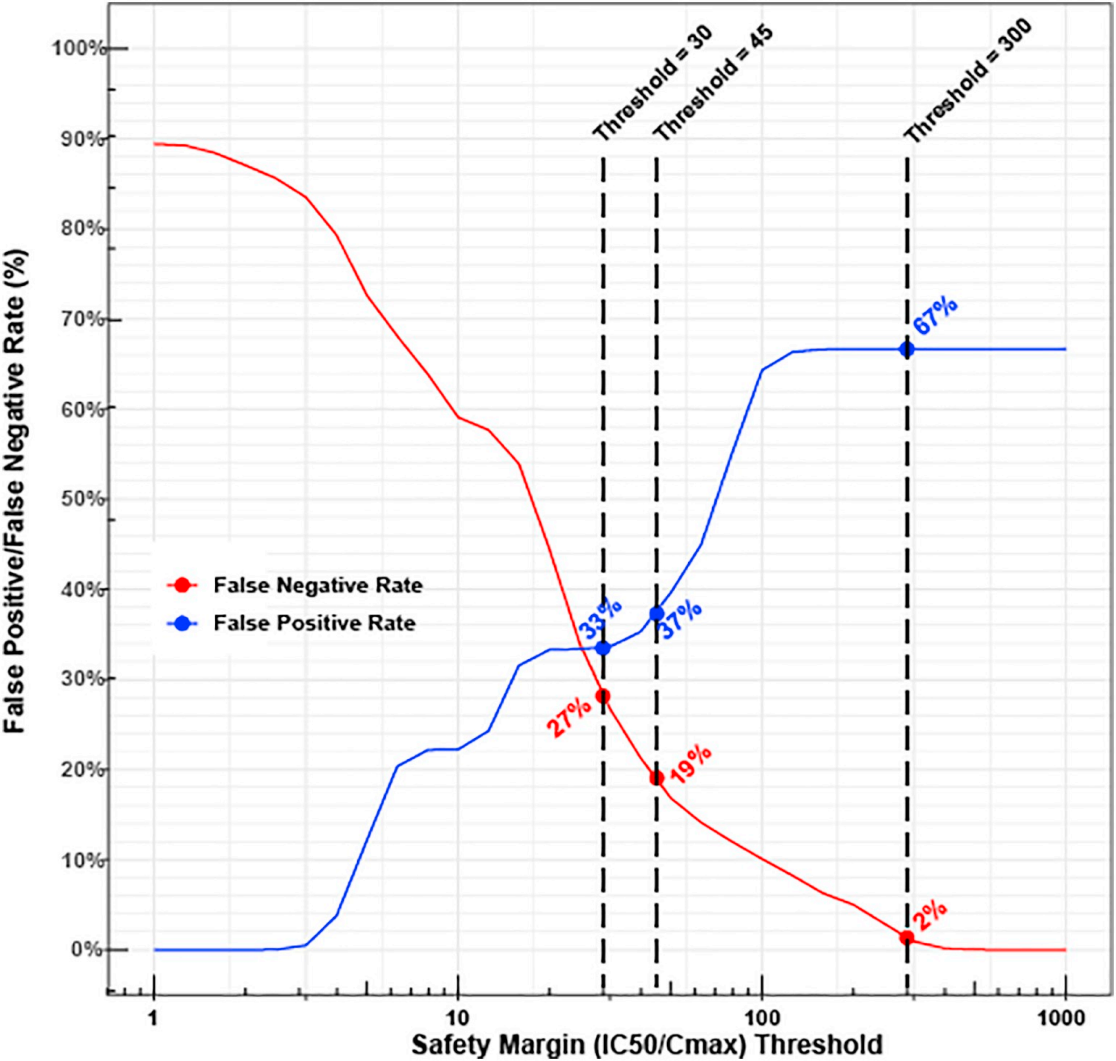
Relationship between the choice of a safety margin (*IC*_50_/*C*_*max*_^*free*^) threshold and the false positive and false negative rates for TdP risk classification. X axis: Any chosen safety margin threshold. Y axis: the false negative (red) and false positive (blue) rates associated with each safety margin threshold. The false positive rate is defined as the probability that a low TdP risk drug will have a safety margin below the threshold. The false negative rate is the probability that an intermediate-risk or high-risk drug will have a safety margin above the threshold. Please see Supplementary Methods for details. All probabilities are based on the posterior probability distributions of *hERG* potency (*IC*_50_) of the 28 drugs divided by corresponding *C*_*max*_^*free*^. Three exemplar thresholds and their associated false positive/negative rates are labeled: A threshold of 300 with very high sensitivity (very low false negative rate) and low specificity (high false positive rate), previously proposed threshold of 45 by Gintant et al. (Gintant, 2011), and the threshold of 30 by Redfern et al. (Redfern et al., 2003). (For interpretation of the references to colour in this figure legend, the reader is referred to the web version of this article.)

**Fig. 3:**
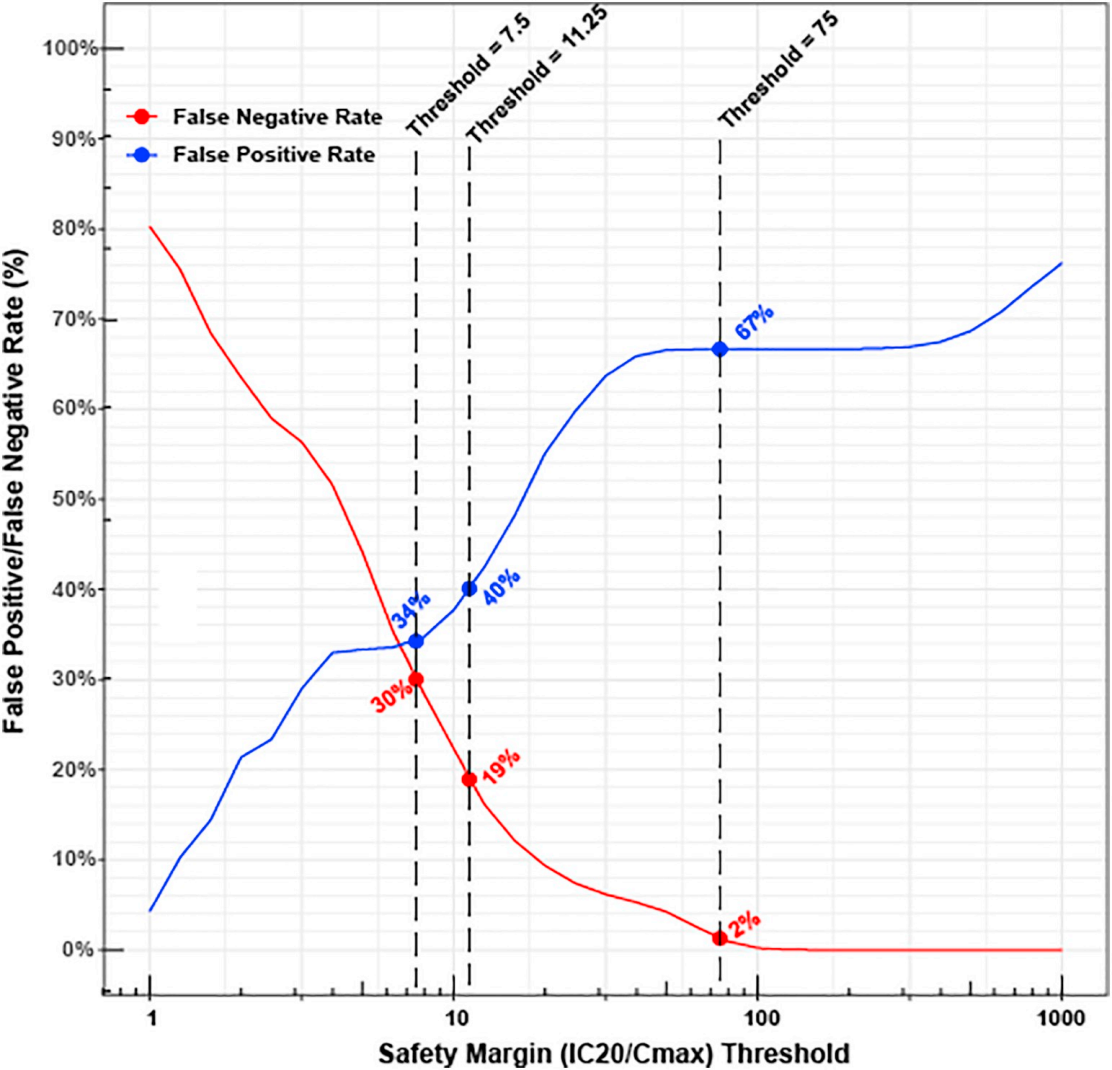
Relationship between the choice of a safety margin (*IC*_20_/*C*_*max*_^*free*^) threshold and the false positive and false negative rates for TdP risk classification. This figure is the same as Fig. 2, but safety margin is defined as *IC*_20_/*C*_*max*_^*free*^ instead of *IC*_50_/*C*_*max*_^*free*^. The three labeled thresholds (75, 11, and 7.5) are scaled from the three *IC*_50_/*C*_*max*_^*free*^-based thresholds (300, 45, and 30) assuming a Hill coefficient of 1.

The high sensitivity of the *hERG* assay can be utilized to define a safety margin threshold to minimize the likelihood of *hERG* block mediated TdP risk, such as the study by Redfern et al. (Redfern et al., 2003)) to define a threshold of 30 by finding an upper bound of *IC*_50_/*C*_*max*_^*free*^ among drugs of considerable TdP liability. A caveat of this approach is that any defined threshold suffers from false positive and false negative rates, and previous studies focused on achieving high sensitivity (low false negative rate) without considering a desired high specificity (low false positive rate). We reasoned that different context of use might place different weights on the tolerability of false positives and false negatives, which would motivate using different thresholds. Accordingly, the relationship between any chosen safety margin threshold and the rates of false positive (probability of low TdP risk drugs having the safety margin below the threshold) and false negative (probability of high or intermediate TdP risk drugs having the safety margin above the threshold) is explored based on the posterior probability distributions of the 28 drugs' *hERG* potency divided by their *C*_*max*_^*free*^ (Li et al., 2017; Li et al., 2018) values (Fig. 2 and Supplementary Methods). We observe the expected trend of a decreasing false negative rate and an increasing false positive rate with increases in threshold. For example, at a threshold of 300, the false negative rate is 2%, but the false positive rate is 67%. When the threshold is moved to 30, the two error rates are closer to each other (false negative and positive rates 27% and 33% respectively). A similar plot is given in Fig. 3 using *IC*_20_/*C*_*max*_^*free*^ as safety margin.

### 3.2 Protocol-dependent *hERG* block potency estimations illustrated by modeling and simulation studies

**Fig. 4:**
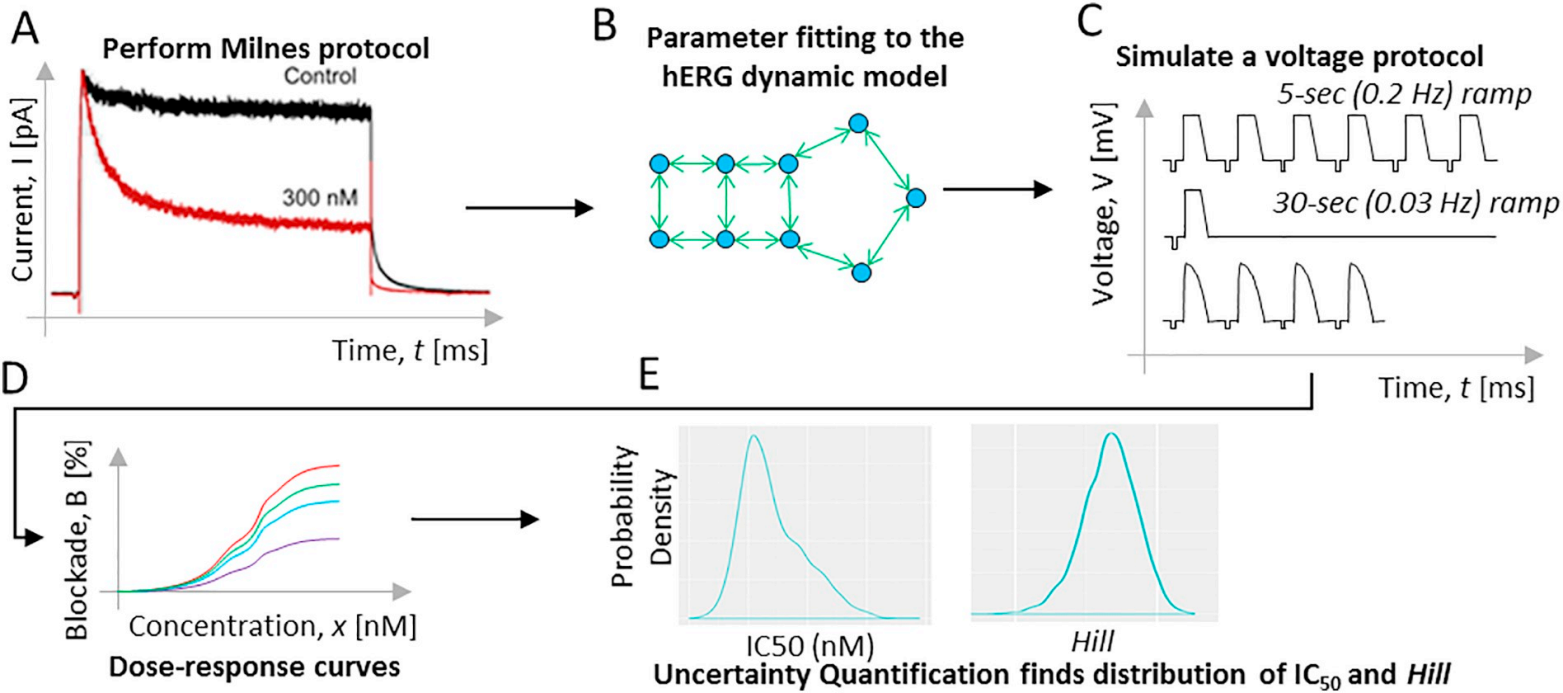
Diagram of using modeling and simulation to illustrate protocol-dependent changes in *hERG* potency estimation. The five panels in Fig. 4 above form a flowchart of the modeling & simulation process to predict protocol-dependent drug block dose response. In panel A, the CiPA dynamic *hERG* protocol (Milnes protocol) was applied to the 28 CiPA drugs. The experimental electrical current (*I*, pA) vs. time (*t*, ms) data were fed into the CiPA *hERG* model (Li et al., 2017) that accounts for drug binding kinetics (panel B) and then used to estimate the *hERG* binding parameters. A bootstrapping procedure (Chang et al., 2017) generates a diverse population of 2000 samples each containing the set of five *hERG* binding parameters. To generate the dose-response curves, the 2000 model parameters were fed into the CiPA *hERG* model, to simulate one of the three voltage protocols (voltage, [*V*, mV] vs. time [*t*, ms]) (panel C). For each protocol, 10 drug concentrations covering a wide range were simulated and the predicted dose-response curves are shown in panel D. Markov-chain Monte Carlo (MCMC) sampling was then used (Chang et al., 2017) to quantify the uncertainty in the dose response curves and generate a credible interval for *IC*_50_ and Hill coefficients (panel E).

One major confounding factor in the *hERG* potency estimation, which is relatively well controlled in this but not in previous studies, is protocol-dependent changes of *IC*_50_s. It has been well established that the voltage protocol and temperature have significant impact on the *hERG* assay results (Kirsch et al., 2004; Lee et al., 2019). But no systematic study has been performed to investigate the effect of different voltage protocols on *hERG* potency estimation. As experimental profiling of such changes across many drugs and different protocols is time consuming, we took advantage of an in silico *hERG* model that has been parameterized by a dynamic protocol to estimate drug binding kinetic parameters (Li et al., 2017). By using this model, we were able to simulate three distinct voltage protocols to predict what potency values we might get from these protocols across all 28 CiPA drugs (Fig. 4). As the *hERG* model was parameterized by dynamic data collected at physiological temperature by a single laboratory, the predicted *IC*_50_s could be regarded as theoretical system-specific *IC*_50_s at 37 °C with intra-site variability from a single site. The three protocols were picked because they represent the standard CiPA *hERG* step-ramp protocol (0.2 Hz) (Li et al., 2018), a slower CiPA step-ramp protocol (0.03 Hz), and an action potential wave form protocol to mimic bradycardia (0.5 Hz) (Sheng et al., 2017). Before using the model for all drugs, a preliminary experiment suggests our model can predict frequency-dependent drug block reasonably well (Supplementary Fig. S2).

A comparison of the predicted *IC*_50_s across the three protocols for all CiPA drugs is shown in Table 2. As expected, different drugs have different change of potency in response to protocol or frequency changes. With the same CiPA protocol, some drugs that are trapped within the closed *hERG* channel during protocol intervals (e.g. bepridil (Li et al., 2017)) have essentially the same *IC*_50_s when varying the frequency from 0.2 Hz to 0.03 Hz. Some highly trapped drugs (e.g. dofetilide (Li et al., 2017)) even have higher block potency (lower *IC*_50_) at slower (0.03 Hz) compared to higher (0.2 Hz) frequency for the same CiPA protocol, illustrating the so called reverse frequency dependent blocking as previously reported (Thomas et al., 2003). By contrast, some less trapped drugs, such as cisapride (Li et al., 2017), have frequency dependent blocking as their *hERG* block potency is higher (the *IC*_50_ is lower) at higher frequency. Other than the tendency of trapping, drug binding rate also plays a role. For instance, terfenadine and ondansetron are two drugs with similar tendency of trapping, and yet quite different binding rates (Li et al., 2017). The slower-binder terfenadine has an over two-fold decrease in block potency (increase in *IC*_50_s) when the CiPA step-ramp protocol frequency is decreased from 0.2 Hz to 0.03 Hz. In contrast the fast-binder ondansetron has almost the same level of block potency when frequencies change. These results show that *hERG* potency estimates depend strongly on the applied voltage protocol. Therefore, any proposed safety margin only has scientific validity when it is in reference to a specific voltage protocol.

**Table 2:** Predicted protocol-dependent *IC*_50_ estimates for the CiPA drugs.

Drug	Risk	Ramp (0.2 Hz) *IC*_50_, [nM]	Ramp (0.03 Hz) *IC*_50_, [nM]	AP (0.5 Hz) *IC*_50_, [nM]
vandetanib	high	199 (197–200)	204 (202–206)	134 (133–135)
sotalol	high	1.095E5 (1.09E5–1.101E5)	1.114E5 (1.106E5–1.121E5)	7.5E4 (7.47E4–7.52E4)
quinidine	high	984 (982–986)	1.099E3 (1.094E3–1.104E3)	624 (623–625)
ibutilide	high	5.47 (5.45–5.49)	3.41 (3.4–3.42)	4.62 (4.6–4.63)
dofetilide	high	9.96 (9.93–9.99)	7.47 (7.45–7.49)	10.36 (10.33–10.38)
disopyramide	high	1.71E3 (1.7E3–1.72E3)	1.99E3 (1.97E3–2E3)	1.248E3 (1.242E3–1.254E3)
bepridil	high	97.3 (97–97.6)	110 (109–111)	70.4 (70.3–70.5)
azimilide	high	257 (256–258)	237 (236–238)	132.7 (132.1–133.3)
terfenadine	intermediate	127.8 (127.2–128.1)	394 (392–395)	54 (53.5–54.4)
risperidone	intermediate	217 (215–219)	627 (622–633)	65 (64–67)
pimozide	intermediate	1.56 (1.53–1.59)	4 (3.9–4.1)	0.92 (0.91–0.93)
ondansetron	intermediate	1.265E3 (1.263E3–1.267E3)	1.31E3 (1.308E3–1.312E3)	967 (966–969)
droperidol	intermediate	164 (163–165)	225 (224–227)	66.5 (65.9–67.1)
domperidone	intermediate	68 (66–69)	75 (71–77)	39.8 (39.4–40.2)
clozapine	intermediate	824 (821–826)	799 (796–802)	690 (689–692)
clarithromycin	intermediate	1.76E4 (1.75E4–1.77E4)	1.78E4 (1.77E4–1.79E4)	1.27E4 (1.26E4–1.28E4)
cisapride	intermediate	23.6 (23.5–23.7)	49.2 (48.8–49.5)	14.5 (14.4–14.6)
chlorpromazine	intermediate	818 (817–819)	777 (776–778)	653 (652–654)
astemizole	intermediate	7.34 (7.29–7.4)	4.3 (4.27–4.33)	6.62 (6.59–6.66)
verapamil	low	620 (616–623)	589 (586–592)	422 (420–423)
tamoxifen	low	553 (550–556)	537 (534–540)	431 (429–432)
ranolazine	low	7.57E3 (7.55E3–7.59E3)	7.62E3 (7.61E3–7.64E3)	6.33E3 (6.32E3–6.34E3)
nitrendipine	low	3.79E4 (3.78E4–3.81E4)	3.86E4 (3.85E4–3.87E4)	3.44E4 (3.42E4–3.45E4)
nifedipine	low	3.67E5 (3.66E5–3.69E5)	3.92E5 (3.9E5–3.93E5)	3.52E5 (3.51E5–3.55E5)
mexiletine	low	1.852E4 (1.849E4–1.855E4)	1.872E4 (1.87E4–1.874E4)	1.733E4 (1.73E4–1.736E4)
metoprolol	low	2.07E4 (2.05E4–2.08E4)	2.09E4 (2.07E4–2.11E4)	2.1E4 (2.08E4–2.11E4)
loratadine	low	6.48E3 (6.44E3–6.51E3)	5.97E3 (5.96E3–5.98E3)	4.225E3 (4.217E3–4.233E3)
diltiazem	low	1.053E4 (1.051E4–1.054E4)	1.088E4 (1.087E4–1.09E4)	1.01E4 (1.009E4–1.011E4)

The CiPA *hERG* model parameterized by Milnes protocol data collected at physiological temperature was used to simulate three protocols and predict dose-response curves for 28 CiPA drugs across multiple concentrations. An uncertainty quantification procedure similar to BHM was used to estimate *IC*_50_s, but with only intra-site variability considered. The 2.5% quantile, 50% quantile, and 97.5% quantiles forming the 95% credible intervals (CI) of *IC*_50_s for all drugs are shown across the three protocols. The unit for all *IC*_50_s is nM. Note that as 2000 simulated cells were used per concentration, the estimated *IC*_50_s generally have lower uncertainty than the variation with protocol dependency. Ramp protocol: CiPA step-ramp protocol. AP protocol: Action potential wave-form protocol. The rationale of selecting these protocols can be found in the Main Text.

## 4 Discussion

In this study, we developed a systematic approach to estimate *hERG* block potency for TdP liability assessment. This strategy aims at addressing four issues associated with previous studies: the use of heterogenous experimental conditions (voltage protocols and temperatures), the ambiguity in the endpoint (TdP vs QTc prolongation), the lack of uncertainty quantification, and the inability to cover those drugs that are difficult to estimate *IC*_50_s due to various reasons such as solubility.

The standardization of experimental protocols was performed as part of a cross-industry HTS automated patch clamp study coordinated by HESI under the CiPA Initiative. The step-ramp protocol was chosen as it is a simple approximation of the shape of a cardiac action potential. Some physiological temperature data were also collected by the HESI study. As most of the data were at ambient temperature, we decided to focus on room temperature data in this project to maximize the coverage of participating sites for a more comprehensive understanding of inter-site variability. To make all sites' experimental conditions as close as possible, not only the voltage protocol but also the sources and concentrations of each compound are “standardized” (stock solutions centrally prepared and then distributed) across sites. A blinding procedure was also implemented to ensure an objective application of the *hERG* assays at each site. The collected multi-site data are an important resource to investigate “why” experimental variabilities exist and identify the most important underlying factors, with the goal of reducing lab-to-lab variability for future *hERG* assays. On the other hand, the collected data also provide a resource to study “how” to use current *hERG* assay data across industry, for example to define a safety margin threshold after considering intra- and inter- site variability. While a manuscript for the former is being prepared, this document represents our effort towards the latter application of the data.

The Bayesian hierarchical modeling approach allows us to quantify intra-site and inter-site variability in a sound statistical framework. A supplementary approach was also developed to define *hERG* block potency using low inhibitory concentrations (*IC*_20_ etc.) to accommodate those drugs with poor solubility or not enough block at highest concentrations. It should be noted that it may be difficult to calculate a reasonable estimate of *IC*_*x*_ if the drug in experimental practice cannot achieve a block of at least *x*. For example, to calculate *IC*_20_ accurately the drug will need to achieve at least 20% block in the *hERG* assay at highest tested concentrations.

Based on the posterior probability distributions of *hERG* potency (*IC*_50_ or *IC*_20_) values as for the 28 drugs derived from BHM, we explored the relationship between safety margin threshold, the false positive rate, and the false negative rate for predicting TdP risk. Similar to previous studies (Redfern et al., 2003; Gintant, 2011) we found that no threshold could achieve both a low false positive rate (high specificity) and low false negative rate (high sensitivity). With a higher threshold (e.g. 300), the high sensitivity (0.98) and low specificity (0.33) give a positive likelihood ratio of 1.5 (a torsadogenic drug is 1.5 times more likely to be classified as torsadogenic compared to a non-torsadogenic drug), and a negative likelihood ratio of 1/16.5 (a non-torsadogenic drug is 16.5 times more likely to be classified as non-torsadogenic compared to a torsadogenic drug), respectively. The lower threshold (e.g. 30) gives a positive likelihood ratio of 2.2, and a negative likelihood ratio of 1/2.5, respectively. The classification performance from either safety margin threshold is lower than the comprehensive in silico model integrating multi-channel pharmacology (Li et al., 2018). This is because the safety margin strategy only considers the *hERG* channel, even though the interplay between multiple ion channels is needed to regulate the action potential. This necessitates the need for comprehensive proarrhythmia risk prediction models that can properly account for the coupled effect of multiple cardiac ion channels. However, under specific context of use, the safety margin strategy could still be utilized. For example, during relatively early drug screening, a lower threshold with a balanced tradeoff between the false positive and false negative rates could quickly screen out many high-risk drugs to produce a “short list” of promising candidates. At later development stage, a higher threshold (lower false negative rate/higher sensitivity) could be applied to determine which drugs have low probability of *hERG* block-mediated TdP liability, and which ones may have enough concern and warrant the use of more comprehensive models for accurate TdP risk assessment.

There are notable limitations to our study. First, although we considered experimental variability of estimating *hERG* block potency in our multi-site study, no uncertainty was associated with *C*_*max*_^*free*^ when calculating the safety margin. This is due to the fact that there are very few studies to establish methods to quantify the uncertainty in the estimation of plasma protein binding, a main factor behind the variability of *C*_*max*_^*free*^ (Wang et al., 2014). Nevertheless, some factors of pharmacokinetic uncertainty did go into our calculation of *C*_*max*_^*free*^. For example, for terfenadine we used a median level of plasma concentration after cytochrome P450 inhibition (Redfern et al., 2003) to account for drug-drug interactions that may be responsible for terfenadine-mediated TdP events. However, not all drugs in this study have enough data to consider drug-drug interactions. In addition, it is known that the metabolite of risperidone, paliperidone, prolongs the QTc interval in a concentration-dependent manner (Suzuki et al., 2012) and that concentrations of this metabolite need to be taken into account for both efficacy and cardiac repolarization. This is analogous to taking in to account the increased exposure of terfenadine occurring in the presence of a metabolic inhibitor, as done in this study. This emphasizes the need to have a good understanding of the distribution, metabolism and elimination of the compounds and any other contributing ion channel effects from metabolites.

A second limitation is that we used model-predicted, rather than experimentally measured, *hERG* block potency across protocols to highlight protocol-dependent changes in *IC*_50_s. We did not attempt to compare the predicted *IC*_50_s from our *hERG* model to experimentally measured *IC*_50_s using the same protocols in the literature, as it will be difficult to dissect the observed differences into intra-site variability and inaccuracy of the model. However, comparing the predicted and observed frequency-dependent *hERG* block data from the same lab suggested that our prediction may have captured the *hERG* dose-response reasonably well. On the other hand, some drugs were predicted by our *hERG* model to have reverse frequency dependency in *hERG* block potency (*IC*_50_s are lower at 0.03 Hz compared to 0.2 Hz with the CiPA step-ramp protocol). Although reverse use dependency on AP prolongation is often observed at the cellular level for many *hERG* blockers (Barandi et al., 2010; Jurkiewicz and Sanguinetti, 1993; Virag et al., 2009), at the channel level drugs are usually observed to have either (forward) frequency dependent or frequency independent block (Stork et al., 2007). Reverse frequency dependency at the channel level is theoretically possible when drugs can bind to the closed *hERG* channel or the tendency to be trapped within the closed *hERG* channel is excessive, but this phenomenon is only occasionally observed for few drugs (Thomas et al., 2003). It is unknown whether our *hERG* model's prediction of more widespread channel-level reverse frequency dependency is due to the protocol selection for the simulation, or theoretical *IC*_50_s with less uncertainty and higher resolution to identify subtle difference, or inaccuracy of the model by overestimating the trapping tendency of some *hERG* blockers. However, this potential discrepancy does not interfere with the model's ability to highlight the pattern of protocol-dependent *IC*_50_ changes. The last and the most important limitation is that, block potency estimations are dependent on data quality and other experimental conditions as well. The HESI multi-site study had a standardized experimental protocol, but no unified quality control criteria. This may affect *IC*_50_ estimates, and subsequently the estimation of the threshold. In addition, a number of experimental conditions were not standardized, and their impact on *hERG* potency estimation is unknown. For instance, different plate types or reservoir materials were used in this study (polypropylene vs glass-coated plates, Teflon reservoirs, glass vials etc.). And while all sites used heterologous cell lines to express human *hERG* channels, different cell types (CHO or HEK293) and biological properties (e.g. channel density) might also contribute to cross-site variability. Our BHM method quantifies the overall inter-lab variabilities from all sources and does not require any specific experimental conditions to be used by different labs (such as heterologous vs iPS or native cardiomyocytes), as long as the underlying experimental protocol is appropriately designed for block potency estimation and standardized across labs. However, understanding the impact of these experimental conditions on potency estimation is essential for future *hERG* assays.

With the proposed establishment of best practice and quality standards for in vitro ion channel studies by the ICH S7B/E14 Q&A process (Questions and Answers, 2018), it is hopeful that in the future similar multi-site studies with not only standard protocol but also unified quality control criteria and other important experimental conditions will be conducted, and then our systematic strategy can be re-applied to the new data to update the *hERG* potency estimation and re-define the safety margin.

## Disclaimer

This report is not an official US Food and Drug Administration guidance or policy statement. No official support or endorsement by the US Food and Drug Administration is intended or should be inferred.

## Declaration of Competing Interest

The authors declared no conflict of interests.

## Appendix A Supplementary data

Supplementary Methods and Data Image 1
