# Corrigendum to “A systematic strategy for estimating hERG block potency and its implications in a new cardiac safety paradigm” [Toxicology and Applied Pharmacology volume 394C (2020) 114961]

**DOI:** 10.1016/j.taap.2020.114983

**Published:** 2020-05-15

**Authors:** Bradley J. Ridder, Derek J. Leishman, Matthew Bridgland-Taylor, Mohammadreza Samieegohar, Xiaomei Han, Wendy W. Wu, Aaron Randolph, Phu Tran, Jiansong Sheng, Timm Danker, Anders Lindqvist, Daniel Konrad, Simon Hebeisen, Liudmila Polonchuk, Evgenia Gissinger, Muthukrishnan Renganathan, Bryan Koci, Haiyang Wei, Jingsong Fan, Paul Levesque, Jae Kwagh, John Imredy, Jin Zhai, Marc Rogers, Edward Humphries, Robert Kirby, Sonja Stoelzle-Feix, Nina Brinkwirth, Maria Giustina Rotordam, Nadine Becker, Søren Friis, Markus Rapedius, Tom A. Goetze, Tim Strassmaier, George Okeyo, James Kramer, Yuri Kuryshev, Caiyun Wu, Herbert Himmel, Gary R. Mirams, David G. Strauss, Remi Bardenet, Zhihua Li

**Affiliations:** a Division of Applied Regulatory Science, Office of Clinical Pharmacology, Office of Translational Sciences, Center for Drug Evaluation and Research, Food and Drug Administration, 10903 New Hampshire Ave, Silver Spring, MD 20993, USA; b Department of Toxicology and Pathology, Eli Lilly and Company, Indianapolis, IN, USA; c Clinical Pharmacology & Safety Sciences, Biopharmaceuticals R&D, AstraZeneca, Cambridge, United Kingdom; d CiPA LAB, 900 Clopper Rd, Suite 130, Gaithersburg, MD 20878, USA; e NMI-TT GmbH, Markwiesenstr. 55, 72770 Reutlingen, Germany; f Sophion Bioscience A/S, Baltorpvej 154, 2750 Ballerup, Denmark; g B'SYS GmbH, The Ion Channel Company, Benkenstrasse 254, CH-4108 Witterswil, Switzerland; h F. Hoffmann-La Roche AG, F. Hoffmann-La Roche Ltd., Bldg. 73 / R. 103b Grenzacherstrasse, 124, CH-4070, Basel, Switzerland; i Eurofins Scientific, Eurofins Discovery, 6 Research Park Drive, St. Charles, MO 63304, USA; j Bristol-Myers Squibb Company, Discovery Toxicology, Bristol-Myers Squibb, 3551 Lawrenceville, Princeton Rd, Lawrence Township, NJ 08648, USA; k Merck & Co., Inc., Kenilworth, NJ, USA; l Metrion Biosciences Limited, Riverside 3, Suite 1, Granta Park, Great Abington, Cambridge CB21 6AD, United Kingdom; m Nanion Technologies Munich, Ganghoferstrasse 70A, 80339 Munich, Germany; n Nanion Technologies USA, 1 Naylon Place, Suite C, Livingston, NJ 07039, USA; o Charles River Laboratories, 14656 Neo Parkway, Cleveland, OH 44128, USA; p Bayer AG, RD-TS-TOX-SP-SPL1, Aprather Weg 18a, 42096 Wuppertal, Germany; q Centre for Mathematical Medicine & Biology, School of Mathematical Sciences, University of Nottingham, Nottingham, United Kingdom; r Université de Lille, CNRS, Centrale Lille, UMR 9189 - CRIStAL, Villeneuve d'Ascq, France

The authors regret that one affiliation address is mistaken in the published paper. Matthew Bridgland-Taylor's affiliation was incorrectly listed as *Clinical Pharmacology & Safety Sciences, R&D, AstraZeneca, Cambridge, United Kingdom*. The correct affiliation is *Clinical Pharmacology & Safety Sciences, BioPharmaceuticals R&D, AstraZeneca, Cambridge, United Kingdom.*

The authors would like to apologise for any inconvenience caused.

