# Supplementary material for: A systematic strategy for estimating hERG block potency and its implications in a new cardiac safety paradigm
Source: Toxicol Appl Pharmacol. 2020 May 1;394:114961. doi: 10.1016/j.taap.2020.114961 (PMC7166077; doi:10.1016/j.taap.2020.114961)
Supplement: Supplementary file 1 — Supplementary Methods and Data [file mmc1.docx]

# Supplementary Materials

## Supplementary Methods

### Electrophysiology

## NMI

The experiments were performed with a QPatch 16 (Sophion, DK) high throughput patch clamp platform with the hERG DUO cell Line from B’SYS GmbH. The composition of the external buffer used was: NaCl 145 mM, KCl 4 mM, D-Glucose 10 mM, MgCl_2_ 1 mM, HEPES 10 mM, CaCl_2_ 2 mM, and a pH of 7.4. The internal buffer was: KCl 120 mM, CaCl_2_ 5.3 mM, MgCl_2_ 1.75 mM, HEPES 10 mM. The test articles are dissolved in: DMSO (SIGMA D8418, Lot #SHBH4245V, Molecular Weight 78.13, purity > 99.9 %), stored in glass vials in darkness. Final testing dilutions where prepared immediately preceding the QPatch run and stored in glass inserts of a QPatch compound plate.

## Sophion Bioscience

A QPatch 48 HTX high throughput patch clamp platform was used with the CHO hERG cell line from B’SYS GmbH (Witterswil, Switzerland). Buffers followed the CiPA instructions, and E-4031 was used at the end of the experiment. Verapamil was used as an internal control. Data was analyzed using the Sophion assay software.

## B’SYS GmbH

The temperature was between 20 ^o^C and 25 ^o^C. Data were recorded on either a Q-Patch 16-well or Q-Patch 48-well HTX high throughput patch clamp platform (single hole chips) using the B’SYS CHO hERG DUO cell line (Passage numbers 12 to 18). Cells were used 3-4 days after plating. Solutions were used as described in the CiPA protocol, with the exception, that 10 mM glucose instead of 11 mM was used in extracellular solution. Cells were stimulated like described in the CiPA protocol. All compounds were tested in at least triplicates; a maximum of 5 cells per compound were tested. Each test solution was freshly prepared by direct dilution of the stock solution following the provided protocol. Increasing test concentrations were perfused to record cumulative dose response curves. Cells were exposed to each test concentration for at least 35 pulses (175 s) at room temperature. At the end of each experiment 1 µM E-4031 was perfused. Only one concentration was tested per cell for the drugs ibutilide, nitrendipine, pimozide, vandetanib, astemizole and tamoxifen. This was done because slow on-rates were observed during pre-experiments. For analysis the current amplitude in the presence of 1 µM E-4031 was subtracted from all current amplitudes before normalization to the current amplitude under control conditions (bath solution only). Data were not run-down corrected, seal resistance was always higher 300 MΩ. For quality control purposes, we required a seal resistance > 300 MΩ during the entire experiment and an initial peak current amplitude > 300 pA.

## Eurofins

Experiments were done with a QPatch single hole high throughput patch clamp platform at 24 ± 2 ^o^C. The cell line used was CHO hERG, catalog number CYL8038, passage P3-P18. Cells were lifted using Accutase^TM^, and re-suspended in CHO-SFMII before the assay.
Test compound(s) were prepared in DMSO to concentrations that were 300x the final assay concentration(s) as described in the project proposal. All 300x DMSO stock solution(s) was transferred to a master plate and from there into assay plates where 1 μL per well of each 300x solution were placed. All assay plates were stored at -80 °C until the day of assay. On the day of the assay, the appropriate assay plate was thawed at room temperature, centrifuged, and 299μl of external solution was added and mixed thoroughly. This provided a 1:300 dilution in total. There was only one freeze thaw cycle of compound plates and glass-coated plates were used for compound storage.

The internal buffer solutions for the hERG assays were: 70 mM KF, 60 mM KCl, 10 mM HEPES, 11 mM EGTA, 10 mM NaCl, 4 mM AGTP, and a pH of 7.3 adjusted using KOH. The external buffer solutions for the hERG assays were: 137 mM NaCl, 4 mM KCl, 1 mM MgCl_2_, 1.8 mM CaCl_2_, 10 mM HEPES, 10 mM glucose, and a pH of 7.35 adjusted using NaOH.

Four test article concentrations were applied sequentially (without washout between test substance concentrations) in ascending order, to each cell. Peak test pulse current was measured during the test ramp. Each concentration was applied for at least 5 minutes before applying a new test article or positive control concentration. At the end of the experiment a supramaximal concentration of a 300 nM E-4031 was applied to assess leak current and appropriate responsiveness of the test system.

A separate group of cells were recorded for each experimental day to determine the baseline response to vehicle and to address day-to-day variability of positive control response. Positive and negative controls were run on each plate to estimate the sensitivity of the system during each run.

Leak current were measured after applying a saturating concentration of a blocker such as E-4031 (300 nM) at the end of each experiment to completely block hERG current. The remaining current, if any, was subtracted from current records. The last 15 seconds of each test concentration data (steady state) were used to determine the dose response relationships. Concentration-response data were fit to an equation of the following form:

$$B=100\times\left[ 1-\frac{1}{\left( 1+\left( \frac{x}{{IC}_{50}} \right)^{h} \right)} \right]$$

Where $B$ is the block, $x$ is the concentration of drug, ${IC}_{50}$ is the concentration of the test article yielding 50% block, and $h$ is the Hill coefficient.

The following quality control parameters were used to rule out the cells that did not meet the criteria.

1. Membrane resistance ($R_{m}$) ≥ 200 MΩ as estimated for a single-cell patch clamp experiment.
2. Leak current ≤ 25% peak current.
3. Baseline current ≥ 0.2 nA.
4. Time-matched vehicle control that matched the addition of the 4 concentrations should be ≤ $0.2\times h$.

## Bristol-Myers Squibb

Experiments were done at 22 ^o^C on an 8-well Patchliner platform (Nanion Technologies, 2 plates per experiment). Stock solutions were prepared with DMSO. The internal buffer solution was 20 mM EGTA, 10 mM HEPES, 50 mM KCl, 10 mM NaCl, 60 mM KF, 285 mOsm, pH 7.2. Most of reagents were purchased from Sigma. The testing compounds were provided by CiPA and stored at -18 ^o^C. The external buffer was: 10 mM HEPES, 140 mM NaCl, 5 mM glucose, 4 mM KCl, 2 mM CaCl_2_, 1 mM MgCl_2_, pH 7.4, 295-305 mOsmol/kg. The cell line used was from Chantest, human Kv11.1 K^+^ ion channel-expressing, stable replicating cell line (HEK), passage P4-P12.

Resistances of the planar patch plate chambers (holes) were 1.8-2.2 MΩ. The cells with membrane resistance ($R_{m}$) <200 MΩ were excluded in the data analysis. Onset and steady state block of hERG current was measured using a pulse pattern, repeated every 5 sec, consisting of a depolarization to 40 mV amplitude for a 500 ms duration, followed by a ramp (1.2 V/s) to -80 mV for 100 ms. The holding potential was -80 mV. Peak tail current was measured during the ramp. Following 5 min recordings with no drug application (baseline), ascending concentrations of the drug were applied for 5 min per concentration. Leak current was measured after applying a saturating concentration of a blocker such as E-4031 (0.5 µM) at the end of each experiment to completely block hERG current. The remaining current was subtracted from current records.

## AstraZeneca

Experiments were performed on the SyncroPatch 384PE (Nanion Technologies) high throughput patch clamp platform at between 22 and 24 °C using 24-well Teflon Reservoirs and medium resistance chips with 4 patch holes per site. hERG-expressing Chinese hamster ovary K1 (CHO) cell line [1] were used in assay-ready format and kept in liquid nitrogen until use. 2 vials of cells were thawed and added to 20 ml external patch clamp solution prior to use on SyncroPatch 384PE. The internal patch clamp solution was KF 120 mM, KCl 20 mM, HEPES 10 mM, EGTA 10 mM, and 25 µM Escin. The external patch clamp solution was NaCl 80 mM, KCl 4 mM, HEPES 10 mM, CaCl2 2 mM, MgCl2 1 mM, glucose 5 mM, and NMDG 60 mM. All the solution reagents were bought from Sigma and stored at room temperature, except Escin, which was stored at 4 °C. All CIPA test compounds and full block compound E-4031 were stored at -20 °C and prepared in glass vials prior to transfer to 24-well Teflon Reservoirs. 0.5 µM E-4031 (Tocris) was added at the end of each experiment to establish a “no current” level. Only wells that passed previously agreed acceptance criteria for this platform were used in this analysis.

## MSD

Frozen CHO-hERG (EMD Millipore #CYL3038) cells were kept in liquid nitrogen until use. A vial of cells was thawed in a 37 °C water bath for 2 minutes and then resuspended in 10 mL hERG external solution. Cells were spun down at 1300 rpm for 75 sec. The cell pellet was resuspended in 500 µL hERG external solution before placing into the QPatch. External solution contained 132 mM NaCl, 4 mM KCl, 3 mM CaCl_2_, 0.5 mM MgCl_2_, 10 mM HEPES, and 11.1 mM glucose (pH 7.35). Internal solution contained 70 mM KF, 60 mM KCl, 5 mM HEPES, 5 mM EGTA, and 15 mM NaCl (pH 7.2). Whole-cell currents were measured from cells using the Sophion QPatch automated patch clamp system and QPLATE 48 single hole plates (Sophion Bioscience A/S, DM) at 22 °C maintained by an attached QPatch heating unit (Sophion QT) and a separate cooling unit (CoolCube, MaxPower Co., CO).  Resistances of the planar patch plate chambers (holes) were 1 ~ 3 MΩ.  Once whole cell conditions were obtained, a 10-minute equilibration period was initiated, after which the vehicle control solution (0.1% or 0.3% DMSO) was added and recording initiated. Voltage-clamp current was low pass filtered with a cutoff of 3 kHz and sampled at 10 kHz. Series resistance was compensated by 80%. An 8 to 10-minute recording was required until the baseline control current stabilized. Subsequent additions of blinded compound concentrations used 3 x 10 μL repeat additions at each concentration.  A cumulative concentration-response curve was obtained by applying four predetermined rising concentrations, each of 5-minute duration, followed by a saturating concentration of 9 µM cisapride to obtain the zero current level during the repolarization ramp. Only cells that met acceptance criteria, as set out by the CIPA Ion Channel Working Group, were included in the final analysis.

## Metrion Biosciences

The ability of test compounds to inhibit the human ether-á-go-go related gene (hERG) potassium channel (Kv11.1) expressed in a Chinese hamster ovary (CHO) cell line was studied using conventional whole-cell patch clamp methodology on the QPatch HT platform (Sophion Bioscience A/S, Denmark). All recordings were made at room temperature (18 ^o^C to 21 ^o^C) using standard single hole chips (1.5 - 4 MΩ). Series resistance (4 - 15 MΩ) was compensated by >80 %. Following establishment of the whole-cell configuration, vehicle was applied to each cell in two bolus additions with a two-minute recording period between each addition. This time period allowed stable recordings to be achieved (four minutes total recording time). Following the vehicle period, a single concentration of test sample was applied as five bolus additions per test concentration at two-minute intervals, after this period verapamil (10 μM), a reference hERG blocker, was applied to yield full inhibition of the current. Currents were elicited using the CiPA voltage protocol and the recording solutions used were those developed in-house to produce < 1% rundown per minute in experiments lasting > 20 minutes. Test compounds were also prepared as serial dilutions in 100% DMSO according to the CiPA method, with 1:333 dilutions of stock into extracellular buffer (0.3% DMSO final).

## Nanion Technologies, Germany

## SyncroPatch 384PE

**Platform**. For this study the 384-well based automated Patch Clamp System SyncroPatch 384PE from Nanion Technologies with PatchControl software (data acquisition) and DataControl software (data analysis) was used. The recordings were performed at room temperature (23°C) on planar NPC-384 multi-hole chips with 4 holes per well at a medium resistance.

**Cells / solutions**. For phase I the stable expressing HEK293 hERG cell line, provided by Chantest (cat # CT6001) was used. The recordings were executed in perforated patch mode applying 25mM Escin with the intracellular solution. The composition of the internal solution was: 120 mM KF, 20 mM KCl, 10 mM HEPES, 10 mM EGTA, pH 7.2 (KOH), 285 ± 3 mOsm. The composition of the external solution was: 80 mM NaCl, 60 mM NMDG, 10 mM HEPES, 4 mM KCl, 2 mM CaCl_2_, 1 mM MgCl_2_, 10 mM Glucose, pH 7.4 (NaOH), 289 ± 3 mOsm, 0.3% DMSO. Phase II compounds were investigated using the stable expressing CHO hERG DUO cell line from by B’SYS GmbH. The recording mode was perforated patch achieved by 25mM Escin in the internal solution. The composition of the internal solution was: 110 mM KF, 10 mM NaCl, 10 mM KCl, 10 mM HEPES, 10 mM EGTA, pH 7.2 (KOH), 280 ± 3 mOsm. The composition of the external solution was: 80 mM NaCl, 60 mM NMDG, 10 mM HEPES, 4 mM KCl, 2 mM CaCl_2_, 1 mM MgCl_2_, 10 mM Glucose, pH 7.4 (NaOH), 289 ± 3 mOsm, 0.3% DMSO. All chemicals were purchased from Sigma Aldrich.

**Compound preparation**. For both phases (I+II) the compounds were solved in 100% DMSO and single-use-aliquots were stored in brown glass vials, protected from light, at -18°C. At the day of experiment a serial dilution in DMSO was prepared manually for all compounds. The pre-diluted compounds were further diluted into external solution with a dilution factor of 1:333.3 (0.3% DMSO). Glass vials were used during compound preparation. Prior experiment execution the compounds were transferred to 24-well Nanion Teflon Reservoirs.

**Compound acquisition mode**: single application, concentrations across the chip. Every well received one compound concentration followed by a fullblock to assess the leak current. Different concentrations of each compound to generate individual dose response relationships were spread across the chip. Besides positive and negative controls on each chip up to five compounds at four concentrations were tested on one chip. Negative control wells were investigated to address rundown / run-up as well as day-to-day variability.

**Electrophysiology.** Cells were stimulated as described in the CiPA recommended guidelines. A voltage protocol consisting of a depolarization step to 40 mV for 500 ms, followed by a ramp (-1.2 V/s) to -80 mV for 100 ms (holding potential -80 mV) was repeated every 5s. A small hyperpolarizing voltage step from -80 to -90 mV was implemented during holding potential to calculate the resistance according to Ohm’s law for quality control (if needed). The peak amplitude of hERG outward tail currents was measured during the repolarizing ramp and normalized to the leak current after applying a saturating concentration of a specific hERG blocker at the end of each experiment.

**QC**. For quality control, the CiPA recommended QC standards were followed:

- Membrane resistance ($R_{m}$) must have been ≥ 200 MΩ as estimated for a single-cell patch clamp experiment for the phase I drugs. Phase II drugs required $R_{m}$≥ 500 MΩ. Because 4x multi-hole chips were used the requested resistances were adapted to $R_{m}$≥ 50 MΩ for phase I and ≥ $R_{m}$125 MΩ for phase II to maintain a comparable seal QC.
- The leak current must have been ≤ 25% peak current.
- The baseline current must have been ≥ 0.2 nA.

## Patchliner

**Platform**. An automated 8-channel Patchliner from Nanion Technologies with PatchControlHT software (to control the Patchliner) and PatchMasterPro software (to control the HEKA amplifiers) was used to participate in this study. The recordings were performed at room temperature (23°C) on planar NPC-16 multi-hole chips with 4 holes per site at a medium resistance.

**Cells / solutions**. For phase I the stable hERG expressing HEK293 cell line, provided by Chantest (cat # CT6001) was used. The recording mode was perforated patch applying 25 mM Escin with the internal solution. The composition of the internal solution was: 120 mM KF, 10 mM KCl, 7mM NaCl, 10 mM HEPES, 5 mM EGTA, pH 7.2 (KOH), 285 ± 3 mOsm. The composition of the external solution was: 80 mM NaCl, 60 mM NMDG, 10 mM HEPES, 4 mM KCl, 2 mM CaCl_2_, 1 mM MgCl_2_, 10 mM Glucose, pH 7.4 (NaOH), 289 ± 3 mOsm, 0.3% DMSO. Phase II compounds were investigated using the stable expressing CHO hERG DUO cell line from by B’SYS GmbH. The recording mode was perforated patch using 25 mM Escin. The composition of the internal solution was: 110 mM KF, 10 mM NaCl, 10 mM KCl, 10 mM HEPES, 10 mM EGTA, pH 7.2 (KOH), 280 ± 3 mOsm. The composition of the external solution was: 80 mM NaCl, 60 mM NMDG, 10 mM HEPES, 4 mM KCl, 2 mM CaCl_2_, 1 mM MgCl_2_, 10 mM Glucose, pH 7.4 (NaOH), 289 ± 3 mOsm, 0.3% DMSO. All chemicals were purchased from Sigma Aldrich.

**Compound preparation**. For both phases (I+II) the compounds were solved in 100% DMSO and single-use-aliquots were stored in brown glass vials, protected from light, at -18°C. At the day of experiment a serial dilution in DMSO was prepared manually for all compounds. The pre-diluted compounds were further diluted into external solution with a dilution factor of 1:333.3 (0.3% DMSO). Glass vials were used.

**Compound acquisition mode**: cumulative dose response. Dose response relationships for each compound were investigated by treating each well with four concentrations in a cumulative manner (lowest to highest concentrations). A fullblock was applied at the end to determine the leak current. Positive and negative controls were included on a daily base to investigate rundown/run-up as well as the day-to-day variability.

**Electrophysiology.** Cells were stimulated as described in the CiPA recommended guidelines. A voltage protocol consisting of a depolarization step to 40 mV for 500 ms, followed by a ramp (-1.2 V/s) to -80 mV for 100 ms (holding potential -80 mV) was repeated every 5s. A small hyperpolarizing voltage step from -80 to -90 mV was implemented during holding potential to calculate the resistance according to Ohm’s law for quality control (if needed). The peak amplitude of hERG outward tail currents was measured during the repolarizing ramp and normalized to the leak current after applying a saturating concentration of a specific hERG blocker at the end of each experiment.

**QC**. For quality control, the CiPA recommended QC standards were followed:

- Membrane resistance ($R_{m}$) must have been ≥ 200 MΩ as estimated for a single-cell patch clamp experiment for the phase I drugs. Phase II drugs required $R_{m}$≥ 500 MΩ. Because 4x multi-hole chips were used the requested resistances were adapted to $R_{m}$≥ 50 MΩ for phase I and ≥ $R_{m}$125 MΩ for phase II to maintain a comparable seal QC.
- The leak current must have been ≤ 25% peak current.
- The baseline current must have been ≥ 0.2 nA.

## Nanion Technologies, USA

## SyncroPatch 384PE

**Platform**. For this study the 384-well based automated Patch Clamp System SyncroPatch 384PE from Nanion Technologies with PatchControl software (data acquisition) and DataControl software (data analysis) was used to investigate phase I compounds. The recordings were performed at room temperature (23°C) on planar NPC-384 multi-hole chips with 4 holes per well at a medium resistance.

**Cells / solutions**. The stable expressing HEK293 hERG cell line, provided by Chantest (cat # CT6001) was used. The recording mode was perforated patch applying 25mM Escin with the internal solution. The composition of the internal solution was: 120 mM KF, 20 mM KCl, 10 mM HEPES, 10 mM EGTA, pH 7.2 (KOH), 285 ± 3 mOsm. The composition of the external solution was: 80 mM NaCl, 60 mM NMDG, 10 mM HEPES, 4 mM KCl, 2 mM CaCl_2_, 1 mM MgCl_2_, 10 mM Glucose, pH 7.4 (NaOH), 289 ± 3 mOsm, 0.3% DMSO. All chemicals were purchased from Sigma Aldrich.

**Compound preparation**. Compounds were solved in 100% DMSO and single-use-aliquots were stored in brown glass vials, protected from light, at -20°C. At the day of experiment a serial dilution in DMSO was prepared manually for all compounds. The pre-diluted compounds were further diluted into external solution with a dilution factor of 1:333.3 (0.3% DMSO). Glass vials were used during preparation. Prior experiment execution the compounds were transferred to 24-well Nanion Teflon Reservoirs.

**Compound acquisition mode**: single application, concentrations across the chip. Every well received one compound concentration followed by a fullblock to assess the leak current. Different concentrations of each compound to generate individual dose response relationships were spread across the chip. Besides positive and negative controls on each chip up to five compounds at four concentrations were tested on one chip. Negative control wells were investigated to address rundown / run-up as well as day-to-day variability.

**Electrophysiology.** Cells were stimulated as described in the CiPA recommended guidelines. A voltage protocol consisting of a depolarization step to 40 mV for 500 ms, followed by a ramp (-1.2 V/s) to -80 mV for 100 ms (holding potential -80 mV) was repeated every 5s. A small hyperpolarizing voltage step from -80 to -90 mV was implemented during holding potential to calculate the resistance according to Ohm’s law for quality control (if needed). The peak amplitude of hERG outward tail currents was measured during the repolarizing ramp and normalized to the leak current after applying a saturating concentration of a specific hERG blocker at the end of each experiment.

**QC**. For quality control, the CiPA recommended QC standards for phase I were followed:

- Membrane resistance ($R_{m}$) must have been ≥ 200 MΩ as estimated for a single-cell patch clamp experiment. Because 4x multi-hole chips were used the requested single-cell resistances was adapted to $R_{m}$≥ 50 MΩ to maintain a comparable seal QC.
- The leak current must have been ≤ 25% peak current.
- The baseline current must have been ≥ 0.2 nA.

## Charles River Laboratories

Experiments were done using an IonWorks Barracuda^TM^ high throughput patch clamp platform. The cell line used was hKv11.1-HEK293 provided by Charles River Laboratories (Wilmington, MA, catalog number CT6001). Cells were maintained in 100 mm cell culture dishes in DMEM/F-12 media supplemented with 10% fetal bovine serum, 100 U/mL of penicillin G sodium, 100 mg/mL streptomycin sulfate, and Geneticin G418. Prior to experiments, the cells were passed in a medium free of Geneticin. Cell density was 50%-70% confluent at the time of harvest. Cells were harvested by washing with Hank’s Balanced Salt Solution (HBSS) and treatment with Accutase^TM^ (Innovative Cell Technologies, San Diego, CA) solution for 30 minutes. Detached cells were transferred in a 15-mL conical tube and resuspended with addition of 10 mL of HBSS. Then the cells were pelleted at 500 g for 2 minutes, the supernatant was removed, and the cell pellet was resuspended in 10 mL of HBSS. The cell suspension was centrifuged again at 500 g for 2 minutes and the supernatant removed. Finally, the cell pellet was resuspended in 5mL of HEPES-buffered physiological saline (HB-PS): 137 mM NaCl, 4 mM KCl, 3.8 mM CaCl_2_, 1 mM MgCl_2_, 10 mM HEPES, and 10 mM glucose, pH adjusted to 7.4 with NaOH, and osmolarity adjusted to 295 ± 5 mOsm. The final cell density was about 10^6^ cells/mL.

Chemicals used in a solution preparation were purchased from Sigma-Aldrich (St. Louis, MO) and were of ACS reagent grade purity or higher. Stock solutions of test compounds were prepared in DMSO and stored at -20^o^C. For experiments test article concentrations were prepared fresh daily by diluting stock solutions into extracellular solutions (HB-PS buffer) supplemented with 2 mM CaCl_2_. The final solution composition for hERG channel was 137 mM NaCl, 4 mM KCl, 3.8 mM CaCl_2_, 1 mM MgCl_2_, 10 mM HEPES, and 10mM glucose, pH adjusted to 7.4 with NaOH. All test and control solutions contained 0.3% DMSO and 0.01% Kolliphor EL. The test article formulations were prepared in 384-well compound plates using a Cyclone automated liquid handling system (Caliper Corp.; Princeton, NJ). The internal HEPES-buffered solution consisted of 70 mM KF, 70 mM KCl, 5 mM MgCl_2_, 2.5 mM EGTA, and 10 mM HEPES, pH 7.2 adjusted with KOH. A stock solution of Escin (perforating agent) was prepared in DMSO (14 mg/mL) and added to the internal solution at a final concentration of 14 µg/mL.

Recordings were performed on an IonWorks Barracuda™ system (Molecular Devices; San Jose, CA) in Population Patch-Clamp™ (PPC) mode as previously described [2, 3]. The extracellular solution was loaded into the PPC plate wells (11 µL/well) and a cell suspension was added into the wells (9 µL/well). After establishment of a whole-cell configuration (7-min perforation), membrane currents were recorded by IWB on-board patch clamp amplifiers.

Test compound concentrations were applied to te cells (4 wells per concentration). Each application consisted of addition of 20 µL of 2X concentrated test article solution to the total 40 µL of final volume of the extracellular well of the PPC planar electrode. Duration of exposure to each test article concentration was five minutes.

Onset and steady state block of hERG current was measured using the CiPA ramp protocol, repeated every 5 seconds, consisting of a depolarizing test pulse to 40 mV for a 500 ms, followed by 100 ms voltage ramp (1.2 V/s) to the holding potential of -80 mV. Peak current was measured during the ramp.

Data Analysis: All data were uniformly corrected for run-down:

$$B_{corrected}=100\times\left[ 1-\frac{\left( B-PC \right)}{\left( VC-PC \right)} \right]$$

where $VC$ is the mean value of the current inhibition with the vehicle control, and $PC$ are the mean values of the current inhibition with the positive control.

Individual well data were filtered according to electrical criteria and the experiments were accepted based on plate level acceptance criteria.

- Seal Resistance (baseline): $R_{seal}\geq500$ MΩ.
- Current amplitude (baseline): ramp peak current ≥ 0.5 nA.
- $Z'$ factor (assay sensitivity) ≥ 0.5.

$Z'$ factor for each experiment was calculated as:

$$Z^{'}=1-3\left( \frac{\sigma_{VC}+\sigma_{PC}}{|\mu_{VC}-\mu_{PC}|} \right)$$

Where $\mu_{VC}$ and $\sigma_{VC}$ were the mean and standard Deviation values for a vehicle control, $\mu_{PC}$ and $\sigma_{PC}$ were the mean and Standard Deviation values for a positive control (3 µM cisapride).

## Bayer AG

The whole-cell voltage-clamp technique (automated 8-channel system: Patchliner, Nanion, Germany) was used with PatchControlHT software (Nanion) to control the Patchliner system and to handle data acquisition and analysis. Voltage-clamp control was provided by two EPC 10 quadro amplifiers under control of the PatchMasterPro software (both: HEKA Elektronik, Lambrecht, Germany). Recordings were done at room temperature using NPC-16 chips (4-hole, ~1 MΩ) (Nanion) serving as planar substrate.

In HEK293 cells with stable expression of the KCNH2 (hERG) K^+^ channel (source: Univ. of Wisconsin), hERG-mediated outward tail currents were elicited using a pulse pattern, repeated every 5 seconds, consisting of a depolarization to 40 mV amplitude for a 500 ms duration, followed by a ramp (-1.2 V/s) to -80 mV for 100 ms (holding potential = -80 mV). Peak tail current was measured during the repolarizing ramp. Leak current was measured after applying a saturating concentration of a blocker such as E-4031 (0.5 µM) at the end of each experiment to completely block hKv1.1/hERG current. The remaining current was subtracted from current records.

The composition of extracellular solution was: NaCl 80 mM, NMDG 60 mM, KCl 4 mM, CaCl_2_ 2 mM, MgCl_2_ 1 mM, HEPES 10 mM, glucose 5 mM, DMSO 0.1%, pH 7.4 (NaOH). The composition of the intracellular solution was: KF 120 mM, KCl 10 mM, NaCl 7 mM, HEPES 10 mM, EGTA 5 mM, pH 7.2 (KOH).

Treatment groups. Concentration response relationships for each test article were independently investigated, i.e. each cell received only one test article, but multiple concentrations in cumulative fashion (lowest to highest concentrations). Each concentration response was based on a minimum of four concentrations, with a minimum of three cells used for each concentration. In case of high variability, more cells per concentration were recorded. Prior to drug addition, cells received an addition of vehicle to measure non-test article-related effects such as addition artifacts. At the end of the experiment, a supramaximal concentration of a known inhibitory reference compound (see above) was applied to assess leak current and appropriate responsiveness of the test system. A separate group of cells (n >3) was recorded for each experimental day to determine the baseline response to vehicle and to address day-to-day variability.

Quality control parameters. Valid whole-cell recordings had to meet the following criteria: (i) membrane resistance ($R_{m}$) ≥ 200 MΩ as estimated for a single-cell patch clamp experiment; (ii) leak current ≤ 25% peak current; (iii) baseline current ≥ 0.2 nA.

Summary Table of HTS sites

| **Site Name** | **Platform Name** | **# of Wells** | **Platform Manufacturer** |
| --- | --- | --- | --- |
| NMI | QPatch 16 | 16 | Sophion Bioscience |
| Sophion Bioscience | QPatch 48 HTX | 48 | Sophion Bioscience |
| B'SYS GmbH | QPatch 16 or QPatch 48 | 16 or 48 | Sophion Bioscience |
| Eurofins | QPatch | 48 | Sophion Bioscience |
| BMS | Patchliner | 8 | Nanion Technologies |
| AstraZeneca | SyncroPatch 384 | 384 | Nanion Technologies |
| MSD | Qpatch 48 | 48  single | Sophion Bioscience |
| Metrion Biosciences | Qpatch 48 HT | 48 | Sophion Bioscience |
| Nanion Technologies Germany | SyncroPatch 384 | 384 | Nanion Technologies |
| Nanion Technologies Germany | Patchliner | 8 | Nanion Technologies |
| Nanion Technologies USA | SyncroPatch 384 | 384 | Nanion Technologies |
| Charles River Laboratories | Ionworks Barracuda | 384 | Molecular Devices |
| Bayer AG | Patchliner | 8 | Nanion Technologies |

| **Site Name** | **Cell Line** | **Cell Line Provider** | **Passage number** | **Current density (**pA/pF) | **Temperature (°C)** |
| --- | --- | --- | --- | --- | --- |
| NMI | CHO | B'SYS | P45-P58 | NA | 23 ± 2 |
| Sophion Bioscience | CHO | B'SYS | ≤ P40 | NA | 26 |
| B'SYS GmbH | CHO | B'SYS | P12-P18 | 46.0±3.0 | 22.5 ± 2 .5 |
| Eurofins | CHO | Eurofins | P3-P18 |  | 24 ± 2 |
| BMS | HEK293 | Chantest | P4-P12 | NA | 22 |
| AstraZeneca | CHO | In-house | P11 | NA | 23 ± 1 |
| MSD | CHO | EMD Millipore | P28 | 22 ±1 (vehicle, n=14) | 22-25 |
| Metrion Biosciences | CHO | B’SYS | P20-50 | 30-60 | 19.5 ± 1.5 |
| Nanion Technologies Germany, Phase I (SyncroPatch 384) | HEK293 | Chantest | P3-5 | NA | 23 |
| Nanion Technologies Germany, Phase II  (SyncroPatch 384) | CHO | B'SYS | P2-10 | NA | 23 |
| Nanion Technologies Germany, Phase I (Patchliner) | HEK293 | Chantest | P15-21 | NA | 23 |
| Nanion Technologies USA | HEK293 | Chantest | P12-15 | NA | 23 |
| Charles River Laboratories | HEK293 | Charles River Laboratories | P63 | NA | ambient |
| Bayer AG | HEK293 | U. Wisconsin | P136-P139 | NA | 22-24 |

| **Site Name** | **Seal Resistance (MΩ)** | **comment** |
| --- | --- | --- |
| NMI | >200 | Average dispense speed 75 µl/s. |
| Sophion Biosciences | Phase I  > 200  Phase II  > 500 | Average dispense speed 75 µl/s. Drug incubation time (per concentration): 300s. |
| B'SYS GmbH | > 300 | Twice 5 uL injections per concentration. At least 36 pulses applied per concentration (3 min). |
| Eurofins | > 200 |  |
| BMS | >200 | Drug incubation time (per concentration): 300s. |
| AstraZeneca | > 200MΩ / hole | Dispense speed 5 µl/s. Drug application time (dispense): 8s  Drug incubation time (for each concentration): 300s |
| MSD | >1000 | Drug incubation time (per concentration): ~ 5 min. Two Qpatch injections for each concentration. |
| Metrion Biosciences | >800 | Average dispense speed 75 µl/s. 4 min current stabilization before applying drug |
| Nanion Technologies Germany, Phase I (SyncroPatch 384) | > 200MΩ / hole | Dispense speed 5 µl/s. Drug application time (dispense): 8s  Drug incubation time (for each concentration): 300s |
| Nanion Technologies Germany, Phase II (SyncroPatch 384) | > 500MΩ / hole | Dispense speed 5 µl/s. Drug application time (dispense): 8s  Drug incubation time: 300s. |
| Nanion Technologies, Phase I (Patchliner) | > 200MΩ / hole | Dispense speed 10 µl/s. Drug application time (dispense): 3s. Drug incubation time: individual for each drug/concentration until steady state. |
| Nanion Technologies USA | > 200MΩ / hole | Dispense speed 5 µl/s. Drug application time (dispense): 8s  Drug incubation time: 300s |
| Charles River Laboratories | > 500 |  |
| Bayer AG | > 200 | Dispense speed 4 µl/s. Drug incubation time (for each concentration): 3 min. |

NA: not available.

### Bayesian Hierarchical Model

## Overview of Bayesian Hierarchical Modeling

A Bayesian hierarchical model (BHM) is a type of mixture model used when the phenomena observed naturally fall into ordered categories that form a hierarchy. The ultimate goal is to calculate a numerical approximation of some posterior distribution. It is a composition of different physical models and constitutive equations that define a joint probability distribution over the data and the parameters. Information flows “up” the hierarchy, with the “hyperparameters” that describe the most general phenomena at the top of it all. Since the model is Bayesian, prior information can be added to the model anywhere within the hierarchy.

BHM has been used previously for solving a variety of scientific and engineering problems, such as computer vision, analysis of DNA microarrays, computational linguistics, and astrophysics [4-7]. In 2016, Johnstone et al. applied BHM to uncertainty quantification of ion channel dose-response data [8]. We have applied a similar methodology in this paper for uncertainty quantification of parameters derived from high throughput ion channel screening platforms.

BHM was used to calculate credible intervals on hERG block parameters (inhibitory concentrations and Hill coefficients) from multi-site data collected for 28 drugs with known torsadogenic risk. The hierarchy is similar to that used by Johnstone et al. 2016 [8]. The prior information from the large-scale study of ion channels in Elkins et al. is also the same [9].

We provide the reader with overviews of:

- Bayes’ Theorem.
- How the likelihood function was constructed.
- Markov-chain Monte Carlo, the Metropolis algorithm, and implementation details.
- Follow-up discussion of how it all works together.

## Overview of Bayes’ Theorem

Bayes’ Theorem allows us to calculate the probability of an event while incorporating quantitative prior evidence into the calculation. This result is given by Bayes’ Theorem:

| $P(A\vert B)=\frac{P\left( B \vert A \right)P(A)}{P(B)}$ | (1) |
| --- | --- |

Where $A$ is an event describing some condition on the experimental parameters, and $B$ is an event described in terms of observed data. $P\left( A | B \right)$ is the conditional probability of event $A$ occurring given that $B$ has occurred, and $P\left( B | A \right)$ is the probability of $B$ occurring given that $A$ has occurred. The left-hand side of (1) is known as the “posterior distribution.” We want to find a numerical approximation of the posterior distribution $P(\boldsymbol{\Theta}|\boldsymbol{X})$, where $\boldsymbol{\Theta}$ is the vector of parameters, and $\boldsymbol{X}$ is the vector of all experimental observations (the “data”). In more specific terms, $P(\boldsymbol{\Theta}|\boldsymbol{X})$ is the probability density at some point $\boldsymbol{\Theta}$ in $K$-dimensional parameter space such that the probability of observing any parameter vector $\hat{\boldsymbol{\Theta}}$ satisfying all $K$ inequalities in (2) is approximately:

| $P(\boldsymbol{\Theta}\vert\boldsymbol{X})\Delta\boldsymbol{\Theta} \cong P(\Theta_{1}\leq\hat{\Theta}_{1}\leq\Theta_{1}+\Delta\Theta_{1},\Theta_{2}\leq\hat{\Theta}_{2}\leq\Theta_{2}+\Delta\Theta_{2},\ldots,\Theta_{K}\leq\hat{\Theta}_{K}\leq\Theta_{K}+\Delta\Theta_{K}\vert\boldsymbol{X})$ | (2) |
| --- | --- |

When multiplied by the volume element $\Delta\boldsymbol{\Theta=}\prod_{\boldsymbol{i=1}}^{K} \Delta\Theta_{i}$. Observe that we used the symbol $\cong$ in (2), which is necessary due to using a finite-sized $\Delta\boldsymbol{\Theta}$. Exact equality is attained in the differential limit as the largest element of$\Delta\boldsymbol{\Theta}$ shrinks to zero.

Equation (1) is a generalized form of Bayes’ Theorem, which can be specified for continuous probability distributions as follows:

| $P(A=\hat{\boldsymbol{\Theta}}\vert B=\hat{\boldsymbol{X}})=\frac{P(B=\hat{\boldsymbol{X}}\vert A=\hat{\boldsymbol{\Theta}})P(A=\hat{\boldsymbol{\Theta}})}{\int_{D} P(B=\hat{\boldsymbol{X}}\vert A=\psi)P(A=\psi)}$ | (3) |
| --- | --- |

Where $D$ is domain of the parameter space, and the events $A$ and $B$ now relate to the parameter vector and the data.

The denominator of (3) is a normalization constant known as the “marginal likelihood”, which ensures the total probability is exactly equal to 1. A problem with computing the marginal likelihood is that it often requires the calculation of an intractable integral. In this work, even the simplest case of two sites would require calculation of a 9-dimensional integral, and the worst-case would have a dimensionality of 33. Clearly, we will need a better method for calculating the posterior distribution.

A workaround for this problem is to generate a numerical approximation of $P(\boldsymbol{\Theta}|\boldsymbol{X})$ through random sampling. However, $P(\boldsymbol{\Theta}|\boldsymbol{X})$ is often a non-canonical probability distribution. Typical computer programming languages usually come with functions for random sampling from a uniform or normal distribution, but randomly sampling from some arbitrary distribution requires more finesse. We want to be clear by what we mean by “random sampling.” We desire to generate a series of vectors, $\boldsymbol{\Theta}_{1}$**,** $\boldsymbol{\Theta}_{2}$**, …,** $\boldsymbol{\Theta}_{M}$ such that taken collectively, they are distributed close to $P(\boldsymbol{\Theta}|\boldsymbol{X})$ for sufficiently large $M$. In other words, for many such vectors, we would observe probabilities close to what would be computed by (2) given an appropriately-sized $\Delta\boldsymbol{\Theta}$.

To summarize and ensure we do not lose the reader, the purpose of generating a numerical approximation to $P(\boldsymbol{\Theta}|\boldsymbol{X})$ is the computational intractability of an exact solution. The exact solution requires calculation of the marginal likelihood, which is generally a high-dimensional nested integral. Also, an exact answer is unnecessary, since arbitrary accuracy is attainable by generating $\boldsymbol{\Theta}_{1}$**,** $\boldsymbol{\Theta}_{2}$**, …,** $\boldsymbol{\Theta}_{M}$ using Markov-chain Monte Carlo (MCMC) methods with the Metropolis algorithm.

To use MCMC however, we require a likelihood and prior. A likelihood is can be thought of as a measure of how well a given vector of parameters fits the data, while the prior distribution measures our initial beliefs about the values of these parameters. Multiplied together, they give a function of the parameters, which is proportional to the posterior density function. The likelihood function takes in multi-dimensional parameters and the data, and outputs a scalar value. The greater the product of the likelihood value and the prior probability, the more frequently a given parameter vector should be observed. By sampling $\boldsymbol{\Theta}$ vectors using the Metropolis algorithm, we can eventually generate a Markov chain that asymptotically approaches the posterior distribution in the differential limit of equation (2).

## Construction of the Likelihood Function

We construct the Bayesian likelihood and the prior piece-by-piece, building it up until it describes the probability of observing the entire vector of uncertain parameters being inferred from the data. We work our way up starting from the site-specific parameters, then the mid-level hyperparameters, and then finally the top-level prior parameters. The subscripted indices in this section have the following meanings:

- $j$, the particular site, e.g. “HTS Site A,” or “HTS Site B.”
- $k$, the particular concentration (in nM).

We assume that, given all parameters, the response of a measured blocking value at a particular drug concentration is normally distributed with a mean equal to that predicted by the Hill equation, and a variance, $\sigma^{2}$, identical at each site and concentration for a particular drug.

| $L(B_{jk})=\frac{1}{\sqrt{2\pi\sigma^{2}}}\exp\left[ -\frac{{(B_{jk}-\bar{B}_{jk})}^{2}}{2\sigma^{2}} \right]$ | (4) |
| --- | --- |

Where $\bar{B}_{jk}$ for all repeated experiments at a given site is given by the Hill equation, which is given by:

| $\bar{B}_{jk}=\frac{100}{1+\left( \frac{{IC}_{50}^{j}}{x_{jk}} \right)^{h_{j}}}$ | (5) |
| --- | --- |

Where $x_{jk}$ is the $k^{th}$ drug concentration at site $j$, $B_{jk}$ is the percentage block predicted by the Hill equation, and ${IC}_{50}^{j}$ and $h_{j}$ are the ${IC}_{50}$ and Hill coefficient for site $j$ ($j$ is an index in the term ${IC}_{50}^{j}$, not an exponent). We introduce here a variable transformation that makes calculation less taxing for the computer. Instead of using ${IC}_{50}^{j}$ in our calculations, we use ${pIC}_{50}^{j}$, given by:

| ${pIC}_{50}^{j}=6-\log_{10} {IC}_{50}^{j}$ | (6) |
| --- | --- |

Where ${IC}_{50}^{j}$ in this calculation is expressed in µM. A unit conversion was necessary here in order for our calculations to comport with the units of the prior information in Elkins et al. 2013 [9].

At the next level of the hierarchy, we assume that $p{IC}_{50}^{j}$ is distributed across sites according to a logistic distribution:

| $L({pIC}_{50}^{j})=\frac{\lambda_{j}}{s{(1+\lambda_{j})}^{2}}$ | (7) |
| --- | --- |

Where $\lambda_{j}=\exp\left( -\frac{{pIC}_{50}^{j}-\mu}{s} \right)$, $\mu$ is the location parameter ($\mu\in[-\infty,+\infty]$), and $s$ is the scale parameter ($s>0$). We also assume that $h_{j}$ is distributed according to a log-logistic distribution:

| $L(h_{j})=\frac{\left( \frac{\beta}{\alpha} \right)\left( \frac{h_{j}}{\alpha} \right)^{\beta-1}}{\left[ 1+\left( \frac{h_{j}}{\alpha} \right)^{\beta} \right]^{2}}$ | (8) |
| --- | --- |

Where $\alpha$ is the scale parameter ($\alpha>0$), and $\beta$ is the shape parameter ($\beta>0$).

Lastly, the contribution from the top level of the hierarchy is the likelihood function accounting for the prior information:

| $L\left( \mu,s,\alpha,\beta,\sigma\vert\boldsymbol{X}_{prior} \right)=P\left( \mu\vert\boldsymbol{X}_{prior} \right)P\left( s \vert\boldsymbol{X}_{prior} \right)P(\alpha\vert\boldsymbol{X}_{prior})P(\beta\vert\boldsymbol{X}_{prior})P(\sigma\vert\boldsymbol{X}_{prior})$ | (9) |
| --- | --- |

Where each component of the product in (9) is given by:

| $\begin{matrix} P\left( \mu\vert\boldsymbol{X}_{prior} \right)=\Gamma_{shift}\left( \mu,k_{\mu}=8.126953,\theta_{\mu}=1.403115,\tau=-4 \right) \\ P\left( s \vert\boldsymbol{X}_{prior} \right)=\Gamma\left( s,k_{s}=5.041652,\theta_{s}=0.03711353 \right) \\ \begin{matrix} P\left( \alpha\vert\boldsymbol{X}_{prior} \right)=\Gamma\left( \alpha,k_{\alpha}=4.558403,\theta_{\alpha}=0.3512812 \right) \\ P\left( \beta\vert\boldsymbol{X}_{prior} \right)=\Gamma\left( \beta,k_{\beta}=8.126953,\theta_{\beta}=0.7015621 \right) \\ P\left( \sigma\vert\boldsymbol{X}_{prior} \right)=\Gamma\left( \sigma,k_{\sigma}=10.90805,\theta_{\sigma}=0.6560324 \right) \end{matrix} \end{matrix}$ | (10) |
| --- | --- |

Where $\Gamma(k,\theta)$ is the standard gamma distribution with shape parameter $k$ and scale parameter $\theta$, $\boldsymbol{X}_{prior}$ is prior data not supplied by sites but discovered through the literature in Elkins et al., and $\Gamma_{shift}$ is a gamma distribution shifted $\tau$ units to the left if $\tau<0$ and to the right if $\tau>0$ [9].

The full likelihood function for drug $i$ is given by taking the sequential product of all the individual probability densities over all site indices $j$ and concentration indices $k$. Note that the index $i$ is not part of the sequential product but is specified by which drug is being investigated.

| $L(\boldsymbol{\Theta}_{i}\vert\boldsymbol{X}_{i},\boldsymbol{X}_{prior})=\prod_{j=1}^{N_{i}^{sites}} \prod_{k=1}^{N_{ij}^{conc}} P_{B_{ijk}}P_{{pIC}_{50}^{ij}}P_{h_{ij}}P_{\mu_{i}}P_{s_{i}}P_{\alpha_{i}}P_{\beta_{i}}P_{\sigma_{i}}$ | (11) |
| --- | --- |

Where $\boldsymbol{X}_{i}$ is data specific to drug $i$, $N_{ij}^{conc}$ is the number of concentrations (including repeats) tested for drug $i$ by site $j$, $N_{i}^{sites}$ is the number of sites that provided data on drug $i$, and $\boldsymbol{X}_{prior}$ is the prior information, which is the same for all 28 drugs.

A difficulty with (11) is that the product’s magnitude is likely to be so small that a computer will be unable to distinguish it from zero. This can be remedied by taking the natural logarithm, which generates the “log-likelihood function.” For later mathematical convenience, we also take the negative, and call the new function $J\left( \boldsymbol{\Theta}_{i} \right)$:

| $\begin{matrix} J\left( \boldsymbol{\Theta}_{i} \right)=-\ln\left[ L\left( \boldsymbol{\Theta}_{i} \vert\boldsymbol{X}_{i},\boldsymbol{X}_{prior} \right) \right]=-(A_{i}+B_{i}+C_{i}+D_{i}) \\ A_{i}=\sum_{j=1}^{N_{i}^{sites}} \sum_{k=1}^{N_{ij}^{conc}} \ln\left( P_{B_{ijk}} \right) \\ \begin{matrix} B_{i}=\sum_{j=1}^{N_{i}^{sites}} \ln\left( P_{{pIC}_{50}^{ij}} \right) \\ C_{i}=\sum_{j=1}^{N_{i}^{sites}} \ln\left( P_{h_{ij}} \right) \\ D_{i}=\ln\left( P_{\mu_{i}} \right)+\ln\left( P_{s_{i}} \right)+\ln\left( P_{\alpha_{i}} \right)+\ln\left( P_{\beta_{i}} \right)+\ln\left( P_{\sigma_{i}} \right) \end{matrix} \end{matrix}$ | (12) |
| --- | --- |

Since probabilities are also between 0 and 1 inclusive, and the logarithms of such numbers are always negative, the function value of (12) is guaranteed to be positive.

Markov-chain Monte Carlo requires a starting parameter vector, $\boldsymbol{\Theta}_{0}$ to begin the sampling. In this work, we always used $\boldsymbol{\Theta}_{0}\boldsymbol{=}{\hat{\boldsymbol{\Theta}}}_{MAPE}$, where ${\hat{\boldsymbol{\Theta}}}_{MAPE}$ is the “maximum *a posteriori* estimate” of the parameter vector. It is the multidimensional mode of the posterior distribution. The ${\hat{\boldsymbol{\Theta}}}_{MAPE}$ is a very similar idea to maximum likelihood estimation (MLE), but includes the prior information, $\boldsymbol{X}_{prior}$. The ${\hat{\boldsymbol{\Theta}}}_{MAPE}$ is the solution of the minimization problem:

| $\begin{matrix} \begin{matrix} \min\\ \boldsymbol{\Theta}_{i} \end{matrix} J\left( \boldsymbol{\Theta}_{i} \right) \\ s.t. \\ 1.751\leq{pIC}_{50}^{j}\leq9.174 \\ \begin{matrix} 0.5\leq h_{j}\leq2.0 \\ 0.005\leq\sigma\leq50.0 \\ \begin{matrix} 0.005\leq s\leq0.981 \\ 2.576\leq\mu\leq8.637 \\ \begin{matrix} 0.362\leq\alpha\leq3.602 \\ 1.539\leq\beta\leq16.379 \end{matrix} \end{matrix} \end{matrix} \end{matrix}$ | (13) |
| --- | --- |

The bounds were determined by finding the 99% quantiles of the marginal distributions in 50 different Markov chains generated from 50 different random seeds. The parameters for the 28 drugs are unlikely to take more extreme values outside of these bounds. If the MAPE calculation failed due to an out-of-bounds ${pIC}_{50}^{j}$, then ${pIC}_{50}^{j}$ was set to $6.0$. Minimization of $J$ is equivalent to maximization of $L$ due to the negative sign. The site-specific Hill coefficients, $h_{j}$, were constrained over $[0.5,2.0]$ based on an observation from the study in Elkins et al. [9]. In that work, large data sets of ion channel screening data were fit to statistical distributions. In Table 1 of that work, despite Hill coefficient being constrained over $[0.5,5.0]$ in their methodology, the fitted values of “$\alpha$” always fell between $[0.5,2.0]$, which is in-line with the theory that Hill coefficient should be $\sim1.0$. The $\alpha$-hyperparameter is used to define a log-logistic distribution, and $\alpha$ is exactly equal to the median of the log-logistic distribution. In the context of this work, $\alpha$ is the median value of Hill coefficient for a drug that would be reported over all sites in the limit as $N_{i}^{sites}\to\infty$. Also, the true $J$ function used in this work was $J\left( \boldsymbol{\Theta}_{i} \right)=-2\times\ln\left[ L\left( \boldsymbol{\Theta}_{i} | \boldsymbol{X}_{i},\boldsymbol{X}_{prior} \right) \right]$, as the R package “FME” expects $J\left( \boldsymbol{\Theta}_{i} \right)$ to be of this form [10].

## Markov-chain Monte Carlo, the Metropolis Algorithm, and Implementation Details

Finding an analytic result for $P\left( \boldsymbol{\Theta} | \boldsymbol{X} \right)$ is difficult. An alternative approach is to generate a numerical approximation using random sampling. The Metropolis algorithm, a variation of basic rejection sampling, is a widely-used algorithm for this exact purpose.

The exact details of the Metropolis algorithm have been discussed at length elsewhere [11]. Briefly, a random move in $\boldsymbol{\Theta}$-space, $\boldsymbol{\Theta}_{i+1}$, is proposed away from the current position, $\boldsymbol{\Theta}_{i}$. The distribution from which random moves are drawn is known as the “proposal distribution.” If $L\left( \boldsymbol{\Theta}_{i+1} | \boldsymbol{X}_{i},\boldsymbol{X}_{prior} \right)\geq L\left( \boldsymbol{\Theta}_{i} | \boldsymbol{X}_{i},\boldsymbol{X}_{prior} \right)$, the move is accepted. Otherwise, the move will still be accepted with some probability, but otherwise rejected. In the mathematical details of the Metropolis algorithm, the marginal likelihood always cancels out, and so its entire calculation can be avoided using this method – only a single evaluation of the likelihood function is required for each element of the Markov chain. No intractable integrals need to be calculated.

In this work, the R package “FME” was used, which utilizes an adaptive Metropolis algorithm to efficiently sample the parameter space [10]. The proposal distribution in FME is a multivariate Gaussian distribution with a mean equal to $\boldsymbol{\Theta}_{i}$ (the current position in $\boldsymbol{\Theta}$-space) and a covariance matrix, $\boldsymbol{\Sigma}$. FME requires a matrix or vector of “jump” parameters, which is an indirect method of specifying $\boldsymbol{\Sigma}$. Inputting a vector, $\boldsymbol{u}_{\boldsymbol{jump}}$, will set $\boldsymbol{\Sigma}$ to a diagonal matrix with its diagonal elements equal to $\boldsymbol{u}_{\boldsymbol{jump}}$. We used the “adaptive” feature of the Metropolis algorithm, i.e., the user-provided jump value will be used for the first few iterations (samples of the chain) only. After that, future jumps are made based on the observed covariance matrix of the chain thus far. The covariance matrix was updated every 10 iterations using the chain’s past values. Our method for specifying the initial $\boldsymbol{u}_{\boldsymbol{jump}}$ is as follows (note: the index $i$ below in this context does not refer to the drug being investigated):

1. Compute ${\hat{\boldsymbol{\Theta}}}_{MAPE}$, and set $W_{0}=1$.
2. Set $\boldsymbol{u}_{\boldsymbol{jump}}=W_{i}{\hat{\boldsymbol{\Theta}}}_{MAPE}$.
3. Run the Markov chain with a 10,000 sample burn in and a chain length of 20,000.
4. Does the acceptance ratio, $r_{accept}$, obey $0.30\leq r_{accept}\leq0.90$?
5. If so, keep $\boldsymbol{u}_{\boldsymbol{jump}}$ and continue running the chain until convergence is suggested by the Geweke diagnostic [12], or the length of the chain reaches 500,000, whichever comes first.
6. If $r_{accept}$ is out of bounds, set $W_{i+1}=(1-\alpha)W_{i}$ if $r_{accept}<0.30$, or $W_{i+1}=(1+\alpha)W_{i}$ if $r_{accept}>0.90$, where in this work $\alpha=0.05$. Then return to step 2.

Literature suggests a “good” approximation of $P(\boldsymbol{\Theta}|\boldsymbol{X})$ is attained if $r_{accept}$ is within these bounds, though there is no general agreement in the literature with what constitutes a “good” or “optimal” bracket for $r_{accept}$ [13]. Step 6 is done to either “tighten up” the jump magnitude if the jumps are too big, or “relax” the jump magnitude if the jumps are too small. Typical acceptance ratios obtained in this work for chains that satisfied the bracket on $r_{accept}$ and the Geweke convergence test were about 60% to 75%, and typical values of $W_{i}$ for the terminal value of $i$ were about 1% to 3%.

The reader can visualize the “random walker” as bouncing around the likelihood landscape, generating additional links in the Markov chain wherever it happens to land in $\boldsymbol{\Theta}$-space. Eventually however, the statistical properties of the chain will not change appreciably with increasing $M$. A probability distribution that does not change with time is said to be a “stationary distribution, or “has achieved stationarity.” The Metropolis algorithm will eventually generate a Markov chain of length $M$ whose final $M_{end}$ elements form a stationary distribution. In the limit of large $M$, this stationary distribution of size $M_{end}$ will converge to the desired posterior distribution, $P(\boldsymbol{\Theta}|\boldsymbol{X})$.

How “large” $M$ must be to arrive at a “useful” approximation is a good question. One way of judging an acceptable magnitude of $M$ is by use of one or more “convergence diagnostics,” which are different types of hypothesis tests. A commonly used diagnostic is the Geweke diagnostic, which has been implemented in the R “coda” package [14]. Geweke’s diagnostic performs a significance test between the first 10% and final 50% of the final $M_{end}$ chain elements. If the means are not statistically different within a given level of confidence, then the chain is presumed to have achieved stationarity; otherwise it has not.

In this work, a “burn in” phase was also used to filter non-stationary samples out of the chains tested for convergence. The first 10,000 samples of the chain were discarded, and then 20,000 samples collected. The acceptance ratio was calculated, and if feasible, the algorithm continued using that value of $W_{i}$. Otherwise, $W_{i}$ was adjusted until a feasible acceptance ratio was achieved. Thenceforth, the chain was restarted, and was extended by 20,000 samples until the Geweke diagnostic passed or the total chain length reached 500,000, whichever came first. The final chain link of one block of 20,000 was used as the starting point for the next block of 20,000. The final block of 20,000 samples was thinned at a ratio of 10:1, which means every 10^th^ sample was taken to form the final approximation of the posterior distribution consisting of 2,000 samples. Thinning is a technique known to greatly reduce autocorrelation in a Markov chain.

## Calculating False Positive and False Negative Rates Associated with Different Thresholds Based on the Posterior Probability Distribution of Safety Margin of the CiPA Drugs

The posterior probability distributions of the 28 CiPA drugs’ hERG potency (either ${IC}_{50}$ or ${IC}_{20}$) were converted to safety margin values by dividing by each drug’s maximum free therapeutic concentration ($C_{max,i}^{free}$). This results in 2,000 safety margin values (samples) for each drug. The safety margin of the $i^{th}$drug is calculated using:

| ${SM}_{50}^{i}=\frac{{10}^{6-\mu_{i}}}{C_{max,i}^{free}}$ | (14) |
| --- | --- |

Where $\mu_{i}$ is the marginal distribution of the logistically-distributed $p{IC}_{50}$ location parameter for the Markov chain generated for the $i^{th}$ drug, and $C_{max,i}^{free}$ is the maximum free therapeutic plasma concentration for drug $i$. The false positive rate and false negative rate were calculated for various thresholds by calculating the proportion of samples from low-risk drugs with a safety margin that fell below the threshold, and the proportion of samples from intermediate-risk and high-risk drugs that fell above the threshold. The data set used was averaged from 50 different MCMC runs, generated using 50 different seeds.

### Using the hERG dynamic model to predict protocol-dependent $\boldsymbol{IC}_{\boldsymbol{50}}$ changes

The experimental data for the 28 CiPA drugs in this work was provided by sites all using some type of high-throughput platform at ambient temperature and using the 5-second CiPA ramp voltage protocol. The FDA In Vitro Electrophysiology Laboratory previously performed voltage clamp experiments to generate data for the 9-state Markov model of the hERG cardiac ion channel for all 28 CiPA drugs [15, 16].These data were collected manually at physiological temperature (37 ^o^C, or 98.6 ^o^F), and used the voltage protocol developed by Milnes et al. [17]. A question we want to answer is, “Can experimental data generated from high-throughput platforms be used for estimating drug safety parameters as well as manual data can?” The way to definitively answer this question however, was infeasible, as it would have required the CiPA experimental team to redo the experiments for all 28 CiPA drugs at ambient temperature and using the 5-second ramp protocol. A tentative result however, could be generated by simulating what the CiPA team would have obtained using the hERG model parameterized by previous kinetic data from the same lab.

In our prior work, the covariance matrix adaptive evolutionary strategy (CMAES) implemented in R was used to generate a set of 2,000 parameter vectors for the hERG model [18]. This was done by integrating the nine differential equations and calculating a weighted “fitting error” that included the sum of squares between the experimental and simulation results for fractional block and “trapping phenotype” [19].

In implementation practice, the objective function for fitting error was calculated by inserting values of $\boldsymbol{\Theta}_{hERG}$ into an ODE numerical solver to simulate time- and voltage- dependent change in channel open probability and current. The simulated open probability/current under drug treatment is normalized against that under control conditions to calculate simulated block. The numerical solver used was lsoda in the R package “deSolve,” a fast implementation of various linear multi-step methods that can handle stiff equations [20]. The simulated block is then compared to observed fractional block for calculating the fitting error. The CMAES algorithm eventually arrived at a population of 2,000 hERG parameter sets that could no longer significantly decrease the fitting error.

The Milnes protocol data used for hERG model parameter estimation are essentially the same as previously published for the 28 CiPA drugs [15, 16]. However, to ensure data quality, an additional quality criterium was applied to select only those cells with less than 20% background current, where the background current was measured by applying 0.5 µM E-4031 to the cell at the end of each experiment. The hERG model for each drug was parameterized using the quality-filtered Milnes protocol data. To obtain the results, we simulated the electrophysiological current trace at 37 ^o^C with a variety of three different voltage protocols. Ten concentrations for each drug were simulated, and the predicted fractional block relevant to the simulated control traces was subjected to a MCMC procedure to estimate ${IC}_{50}$s and Hill coefficients under each specific protocol.

To implement this strategy, a code was written in the R programming language, which we term “LabClone,” since its purpose is to simulate, or “clone,” the results the CiPA experimental team would have obtained. LabClone requires several things to work:

- A .csv file for each drug containing the 2,000 hERG parameters as estimated by the Milnes protocol data.
- A set of concentrations to iterate over for each drug.
- A temperature. In this work, it is 37 ^o^C.
- A voltage protocol.
- Intracellular ($\left[ K^{+} \right]_{IC}$) and extracellular ($\left[ K^{+} \right]_{EC}$) potassium ion concentration.
- A cutoff threshold for determination of steady-state current. In this work, it is 0.1%.

In this work, we chose a set of concentrations for each drug that was logarithmically-spaced between a very small value and a very high value. This was to ensure the simulation would reach the highest possible block at the highest concentrations, and make sure the dose-response curve had the expected sigmoidal shape for a plot of fractional block vs. $\log_{10}\left( C \right)$.

The LabClone simulation works as follows:

1. For a given drug, input one of its 2,000 hERG parameter vectors.
2. Simulate the response when no drug is added for the selected voltage protocol.
3. Identify the maximum electrical current over the simulation trace.
4. Set the final state vector as the initial state for the next simulation.
5. Repeat steps 2 through 4 until the obtained maximum electrical current changes from trace-to-trace by 0.1% or less.
6. Repeat steps 2 through 5, but now with drug added at some concentration.
7. Calculate the fractional block.
8. Repeat steps 6 through 7 for all concentrations a given drug is to be simulated with.
9. Repeat steps 1 through 8 for all 28 drugs and all 2,000 of their hERG parameter set samples.

The electrical current is calculated as:

| $I(t,C)=P_{open}\left( t,C \right)\left[ V\left( t \right)-V_{rev} \right]$ | (15) |
| --- | --- |

Where $I$ is the electrical current [pA], $C$ is the drug concentration [nM], $P_{open}\left( t \right)$ is the “open probability” Markov state at time $t$, $V\left( t \right)$ is a function representing the voltage protocol used [mV], and $V_{rev}$ is the reversal voltage [mV]. $V_{rev}$ is calculated as:

| $V_{rev}=\frac{R_{gas}T}{F}\ln\left( \frac{\left[ K^{+} \right]_{EC}}{\left[ K^{+} \right]_{IC}} \right)\times\frac{1000 \mathrm{mV}}{1 V}$ | (16) |
| --- | --- |

Where $R_{gas}$ is the universal gas constant (8.314 J/mol·K), $T$ is the absolute temperature in Kelvin, $F$ is Faraday’s constant (96,485 coulombs/mol), $\left[ K^{+} \right]_{EC}$ is the extracelluar concentration of potassium ions, and $\left[ K^{+} \right]_{IC}$ is the intracellular concentration of potassium ions. In this work, $\left[ K^{+} \right]_{EC}=5$ millimolar, and $\left[ K^{+} \right]_{IC}=150$ millimolar.

Fractional block is calculated as:

| $B\left( C \right)=1-\frac{\max\left[ I_{drug}^{ss}\left( t,C \right) \right]}{\max\left[ I_{control}^{ss}\left( t,C=0 \right) \right]}$ | (17) |
| --- | --- |

Where $I_{drug}^{ss}$ and $I_{control}^{ss}$ are the steady-state electrical current traces (less than 0.1% change in the maximum electrical current value from the last trace or after 100 simulations, whichever came first), and the maximum values over $t$ are used.

While the simulation is large, the problem is amenable to parallelization. Using the FDA’s high-performance computing clusters, cycling through all 2,000 parameters for all 28 drugs takes about three hours.

Performing this calculation results in 2,000 different dose-response curves for each drug. These can then be processed using code similar to the Markov-chain Monte Carlo (MCMC) code discussed in our previous work (but without the higher level hyperparameters to describe inter-site variability, since there is only one site). Two modifications were made to this code. Since the hERG model uses an $E_{\max}$ model to assume a saturating maximum drug effect on the channel [21], the Hill equation was modified to assume a maximum block ($B_{\max}$). This is similar to a modified Hill equation to introduce an $I_{\max}$ effect previously used for analysis of CiPA hERG data [22]. The modified Hill equation has the form:

| $B(x)=\frac{B_{max}}{1+\left( \frac{{IC}_{50}}{x} \right)^{h}}$ | (18) |
| --- | --- |

Where $B_{max}$ is a new parameter that assumes a maximum, or saturating, blocking effect a drug may have on the hERG channel, corresponding to the maximum binding rate ($E_{\max}$) in the dynamic hERG model. In this work, we enforced $70\%\leq B_{max}\leq100\%$. Also, the new likelihood function now explicitly incorporates the variance between observed block and the Hill-predicted mean block.

The general strategy for the MCMC is the same as in our previous work [15]:

1. Compute ${\hat{\boldsymbol{\Theta}}}_{MLE}^{MCMC}$, which is the value of $\boldsymbol{\Theta=}\left[ \begin{matrix} B_{max} & {IC}_{50} & \begin{matrix} h & \sigma^{2} \end{matrix} \end{matrix} \right]^{T}$ obtained using maximum-likelihood estimation.
2. Set ${\hat{\boldsymbol{\Theta}}}_{MCMC}^{MLE}=\boldsymbol{\Theta}_{0}^{MCMC}$, the initial point for the MCMC random sampling.
3. Run the Metropolis algorithm using the same jump selection strategy as discussed in the section on BHM above, and with the same convergence tolerance on the Geweke diagnostic.
4. Repeat steps 1 through 3 for all 28 drugs whose effect was simulated using LabClone.

This generates 2,000 $\boldsymbol{\Theta}^{MCMC}$ parameter vectors sampled from their posterior joint distribution. We are most interested in the marginal distribution for ${IC}_{50}$ and ${IC}_{20}$.

### Nomenclature

| **Symbol** | **Description** | **Scientific Units** | **Value or Domain** |
| --- | --- | --- | --- |
| $P$ | A probability of some event | N/A | $P\in[0,1]$ |
| $P(A\vert B)$ | Probability of event $A$ happening given that event $B$ has occurred | N/A | $P(A\vert B)\in[0,1]$ |
| $P(\boldsymbol{\Theta}\vert\boldsymbol{X})$ | Probability of observing parameter vector $\boldsymbol{\Theta}$ given data set $\boldsymbol{X}$ | N/A | $P(\boldsymbol{\Theta}\vert\boldsymbol{X})\in[0,1]$ |
| $\boldsymbol{\Theta}$ | Parameter vector. In this work, it is the vector of site-specific drug parameters and mid-level hyperparameters | Depends on vector element | Depends on vector elements |
| $\boldsymbol{X}$ | Data sets provided by sites in this work; hERG channel dose response data for the 28 CiPA drugs | Concentration is in nM; block in % | Concentration > 0,  Block is 0% to 100% |
| $K$ | The number of elements in the parameter vector | N/A | $K\in\left[ 9,33 \right]$  9 is if only two sites contributed data; 33 is if the maximum possible value of 14 sites contributed data |
| $\hat{\boldsymbol{\Theta}}$ | Any parameter vector that can satisfy all of the inequalities in equation (2) | Depends on vector element | Depends on vector elements |
| $\Delta\boldsymbol{\Theta}$ | Volume element of the parameter space | Product of all the units in the parameter vector | Depends on vector elements |
| $D$ | The domain of integration in equation (3) | N/A | N/A |
| $\boldsymbol{\Theta}_{1}$**,** $\boldsymbol{\Theta}_{2}$**, …,** $\boldsymbol{\Theta}_{M}$ | A Markov-chain of length $M$ of parameter vectors sampled using the Metropolis algorithm | N/A | N/A |
| $i,j,$ and $k$ | Indices for drug, site, and concentration, in that order, for indexing both the site data and the sampled parameters | N/A | N/A |
| $L$ | The likelihood function | N/A | N/A |
| $\sigma^{2}$ | Variance in observed drug block | N/A | $\sigma^{2}>0$ |
| $B_{jk}$ | Observed fractional block for a drug, measured by site $j$, at concentration $k$ | N/A | $B_{jk}\in[0,1]$ |
| $\bar{B}_{jk}$ | Expected value of the fractional drug block for the same indices | N/A | $\bar{B}_{jk}\in[0,1]$ |
| ${IC}_{50}^{j}$ | ${IC}_{50}$ for a drug obtained by site $j$ | nM | ${IC}_{50}^{j}>0$ |
| $h_{j}$ | Hill coefficient for the same indices | N/A | $0.5\leq h_{j}\leq2.0$ |
| $x_{jk}$ | Experimental concentration for the same indices as above | nM | $x_{jk}>0$ |
| ${pIC}_{50}^{j}$ | A logarithmic transformation of ${IC}_{50}^{j}$ that makes calculation easier for the computer | N/A | Whole real line |
| $\lambda_{j}$ | An auxiliary variable used for simplifying the presentation of equation (7) | N/A | $\lambda_{j}\in[0,1]$ |
| $s$ | The ${pIC}_{50}$ “scale” hyperparameter for a logistic distribution a drug | N/A | $s>0$ |
| $\mu$ | The ${pIC}_{50}$ “location” hyperparameter for a logistic distribution for drug $i$ | N/A | Whole real line |
| $\alpha$ | The $h$ “scale” hyperparameter for a log-logistic distribution a drug | N/A | $\alpha>0$ |
| $\beta$ | The $h$ “shape” hyperparameter for a log-logistic distribution for a drug | N/A | $\beta>0$ |
| $\boldsymbol{X}_{prior}$ | Prior parametric information for distributions describing hERG cardiac ion channel behavior (from Elkins et al. [9] | N/A | N/A |
| $\Gamma(k,\theta)$ | The standard gamma probability distribution with shape parameter $k$ and scale parameter $\theta$. | N/A | $\Gamma\left( k,\theta\right)>0,$  $k>0,$  $\theta>0$ |
| $\Gamma_{shift}(k,\theta,\tau)$ | A shifted standard gamma distribution, which is shifted $\tau$ units to the left if $\tau<0$, and likewise to the right if $\tau>0$ | N/A | $\Gamma_{shift}(k,\theta,\tau)>0$  $k>0,$  $\theta>0$  $\tau=-4$ |
| $k_{\mu}$,$\theta_{\mu}$,$k_{s}$,$\theta_{s}$,$k_{\alpha}$,$\theta_{\alpha}$,  $k_{\beta}$,$\theta_{\beta}$,$k_{\sigma}$,$\theta_{\sigma}$ | Gamma distribution parameters for the prior probability distributions | Depends on variable | $k>0,$  $\theta>0$ |
| $-\ln\left[ L\left( \boldsymbol{\Theta} \vert\boldsymbol{X},\boldsymbol{X}_{prior} \right) \right]$, or $J\left( \boldsymbol{\Theta} \right)$ for simplicity | The “negative log-likelihood function” | N/A | $J\left( \boldsymbol{\Theta} \right)\geq0$ |
| $A,B,C,$ and $D$ | Auxiliary variables used for simplifying the presentation of equation (12) | N/A | Must be less than or equal to zero |
| ${\hat{\boldsymbol{\Theta}}}_{MAPE}$ | Maximum *a posteriori* estimate of the parameter vector | Depends on vector element | Depends on vector elements |
| $\boldsymbol{u}_{\boldsymbol{jump}}$ | The jump vector for making jumps in parameter space when performing MCMC | Depends on vector element | Depends on vector elements |
| $W_{i}$ | Scaling factor multiplied by ${\hat{\boldsymbol{\Theta}}}_{MAPE}$ to calculate $\boldsymbol{u}_{\boldsymbol{jump}}$ | N/A | $W_{i}>0$ |
| $\boldsymbol{\Sigma}$ | Covariance matrix in the multivariate Gaussian proposal distribution used by the R package “FME” | Depends on matrix elements | Main diagonal elements are all greater than zero; off-diagonal elements span the whole real line |
| $r_{accept}$ | The acceptance ratio, defined as the number of times a jump was proposed and was accepted, divided by the chain length | N/A | $r_{accept}\in[0,1]$ |
| $\alpha$ | Adjustment parameter for increasing or decreasing $W_{i}$ depending on if $r_{accept}$ was out-of-bounds | N/A | $\alpha=0.05$ |
| $M_{end}$ | The final elements of the Markov chain used for producing the numerical approximation of the posterior distribution | N/A | $M_{end}=20,000$, but was thinned by taking every 10^th^ element, and thus the final sampled distribution had 2,000 vector elements |
| ${SM}_{50}$ | The safety margin based on ${IC}_{50}$ | N/A | ${SM}_{50}>0$ |
| $C_{max}^{free}$ | The maximum free concentration of drug in blood plasma that would be administered for therapeutic purposes | nM | $C_{max}^{free}>0$ |
| $I(t,C)$ | Cardiac ionic electrical current as a function of time, $t,$and drug concentration, $C$ | pA | Whole real line |
| $P_{open}$ | Probability the ion channel is open | N/A | $P_{open}\in[0,1]$ |
| $V\left( t \right)$ | Voltage as a function of time; it is the voltage protocol | mV | Whole real line |
| $R_{gas}$ | The universal gas constant | $\frac{J}{mol\cdot K}$ | $8.314\frac{J}{mol\cdot K}$ |
| $T$ | Temperature of the voltage clamp experiments | ^o^C in the R code input, but converted to Kelvin for the $V_{rev}$ calculation | 37 *^o^C* or $310.15 K$ |
| $F$ | Faraday’s constant | coulombs per mol | $96,485\frac{C}{\mathrm{mol}}$ |
| $\left[ K^{+} \right]_{EC}$ | Extracellular potassium ion concentration | millimolar | $150 mM$ |
| $\left[ K^{+} \right]_{IC}$ | Intracellular potassium ion concentration | millimolar | $5 mM$ |
| $V_{rev}$ | The reversal potential; the electric potential at which, if applied, lead to a measurement of exactly “zero” for ionic current | mV | $\frac{(8.314)(37+273.15)}{96,485}\ln\left( \frac{5 \mathrm{nM}}{150 \mathrm{nM}} \right)\times\frac{1000 \mathrm{mV}}{1 V}= -39.476 \mathrm{mV}$ |
| $I_{drug}^{ss}$ | Steady state current trace from simulation in the presence of drug, defined as less than 0.1% change in the maximum value | pA | Whole real line |
| $I_{control}^{ss}$ | Steady state current trace from simulation in the absence of drug, defined as less than 0.1% change in the maximum value | pA | Whole real line (depends on voltage protocol) |
| $B_{max}$ | A new parameter in the non-hierarchical MCMC that accounts for low maximum block | % | $B_{max}\in[70\%,100\%]$ |
| ${\hat{\boldsymbol{\Theta}}}_{MCMC}^{MLE}$ | Parameter vector in the non-hierarchical MCMC model obtained using maximum-likelihood estimation | Depends on vector element | Depends on vector elements |

## Supplementary Figures and Tables

**A**

**B**

**
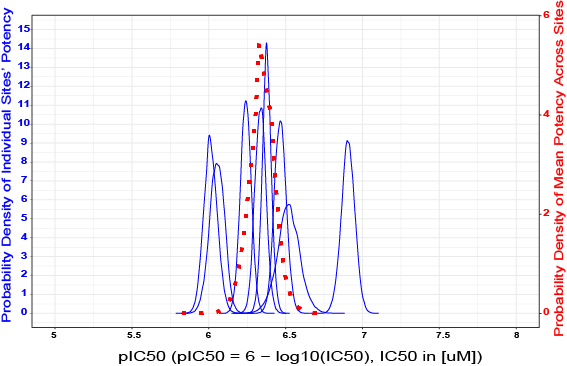
**

**Figure S1**. An example of inter- and intra- site variability.

(**A**) Multi-site dose-response data for azimilide is shown. Each site has its own color for experimental data (dots) and Hill fitting (curves). Points with different colors tend to be separated, showcasing inter-site variability. Points with the same color also have a spread at each concentration, illustrating intra-site variability. (**B**) Marginal probability distributions of azimilide’s hERG block potency as estimated by the Bayesian Hierarchical Model (BHM). The hERG potency is represented as pIC50s, as shown on the X axis. Each site’s dose-response data from (A) were used to derive a distinct probability distribution of pIC50s (blue curves). From all these site-specific distributions, a higher-level logistic distribution is estimated with two hyperparameters: a location parameter $\mu$ and a scale parameter $s$. The location parameter indicates the mean hERG potency estimated across sites, and the scale parameter indicates the variability of hERG potency across sites. The probability distribution of the location parameter for azimilide is shown as the red dotted curve.

###
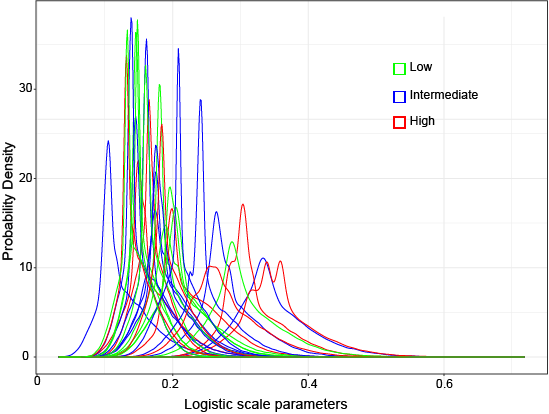


**Figure S2**. CiPA compounds’ cross-site variability in hERG block potency estimates.

The marginal posterior distribution of the scale parameter $s$ of the higher-level logistic distribution, which indicates the variability of the hERG block potency estimates across sites for each drug, is shown for all 28 CiPA compounds. High, Intermediate, and Low risk compounds are represented by red, blue, and green curves, respectively.

###
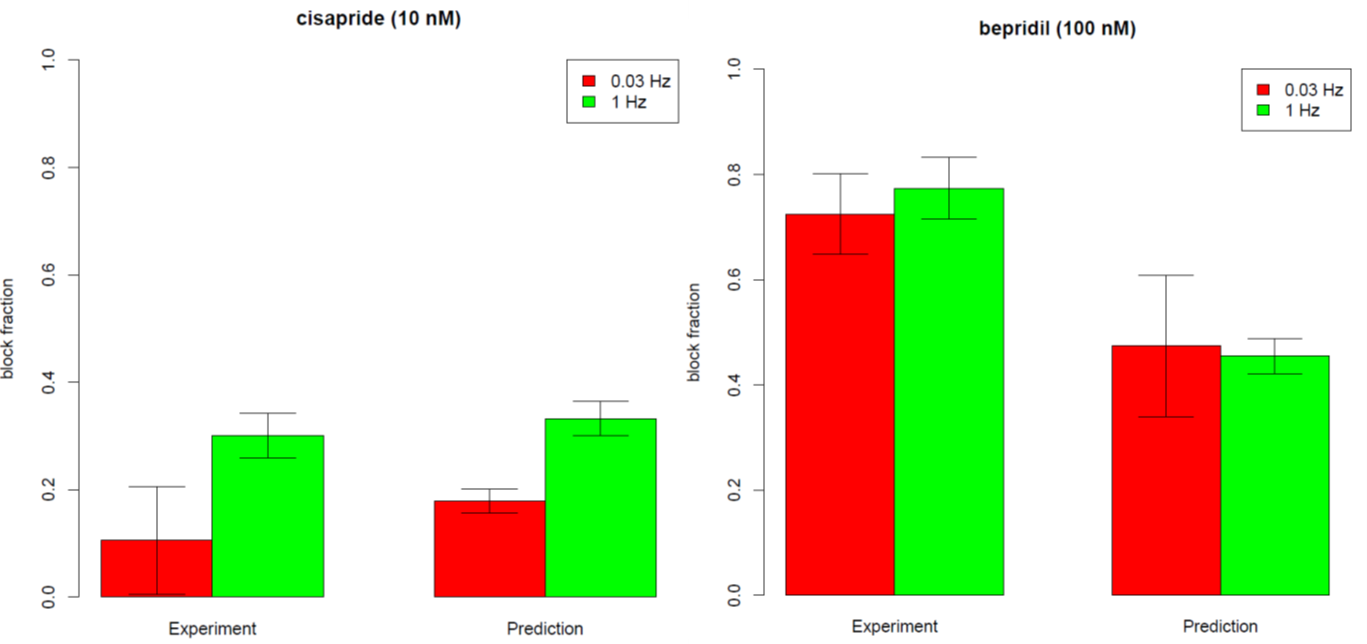


**Figure S3.** Fractional block prediction for cisapride (10 nM) and bepridil (100 nM). The voltage protocol was an experimentally-observed action potential (AP) waveform, either at 1 action potential every 30 seconds (0.03 Hz), or 1 action potential every second (1 Hz). Experimental details of the AP protocol were published previously [23]. The pre-recorded rabbit ventricular AP waveform used as the voltage command in this experiment was kindly provided by Dr. Gail Robertson (University of Wisconsin-Madison) [24, 25]. The dynamic hERG model, after parameterized with cisapride and bepridil under the Milnes protocol at physiological temperature as previously reported, was used to predict the outcome of the action potential waveform protocol [15]. Left Panel: observed (left) and predicted (right) block at 10 nM cisapride using the AP protocol. At higher frequency (1 Hz) cisapride demonstrated higher block compared to lower frequency (0.03 Hz). Such a frequency-dependent block was predicted well. Right Panel: observed (left) and predicted (right) block at 100 nM bepridil using the AP protocol. Bepridil demonstrated frequency-independent block across the two frequencies, which is predicted correctly. Note that the absolute amount of block as predicted is lower than observed, possibly due to the fact that the dynamic hERG model was optimized to replicate the behavior of $I_{Kr}$ current in an integrated cardiomyocyte [19], rather than the simple hERG channel expressed in vitro as used in this experiment.

### References

1. Persson, F., et al., *Blocking Characteristics of hERG, hNav1.5, and hKvLQT1/hminK after Administration of the Novel Anti-Arrhythmic Compound AZD7009.* Journal of Cardiovascular Electrophysiology, 2005. **16**(3): p. 329-341.

2. Wible, B.A., et al., *An Ion Channel Library for Drug Discovery and Safety Screening on Automated Platforms.* ASSAY and Drug Development Technologies, 2008. **6**(6): p. 765-780.

3. A., K.Y., et al., *Evaluating State Dependence and Subtype Selectivity of Calcium Channel Modulators in Automated Electrophysiology Assays.* ASSAY and Drug Development Technologies, 2014. **12**(2): p. 110-119.

4. Broët, P., S. Richardson, and F. Radvanyi, *Bayesian Hierarchical Model for Identifying Changes in Gene Expression from Microarray Experiments.* Journal of Computational Biology, 2002. **9**(4): p. 671-683.

5. Fei-Fei, L. and P. Perona. *A Bayesian hierarchical model for learning natural scene categories*. in *2005 IEEE Computer Society Conference on Computer Vision and Pattern Recognition (CVPR'05)*. 2005.

6. Mandel, K.S., et al., *Type IA Supernova Light-Curve Inference: Hierarchical Bayesian Analysis in the Near-Infrared.* The Astrophysical Journal, 2009. **704**(1): p. 629-651.

7. Teh, Y.W., *A hierarchical Bayesian language model based on Pitman-Yor processes*, in *Proceedings of the 21st International Conference on Computational Linguistics and the 44th annual meeting of the Association for Computational Linguistics*. 2006, Association for Computational Linguistics: Sydney, Australia. p. 985-992.

8. Johnstone, R.H., et al., *Hierarchical Bayesian inference for ion channel screening dose-response data.* Wellcome open research, 2017. **1**: p. 6-6.

9. Elkins, R.C., et al., *Variability in high-throughput ion-channel screening data and consequences for cardiac safety assessment.* Journal of Pharmacological and Toxicological Methods, 2013. **68**(1): p. 112-122.

10. Soetaert, K. and T. Petzoldt, *Inverse Modelling, Sensitivity and Monte Carlo Analysis in R Using Package FME.* 2010, 2010. **33**(3): p. 28.

11. Beichl, I. and F. Sullivan, *The Metropolis Algorithm.* Computing in Science & Engineering, 2000. **2**(1): p. 65-69.

12. Cowles, M.K. and B.P. Carlin, *Markov Chain Monte Carlo Convergence Diagnostics: A Comparative Review.* Journal of the American Statistical Association, 1996. **91**(434): p. 883-904.

13. Foreman-Mackey, D., et al., *emcee: The MCMC Hammer.* Publications of the Astronomical Society of the Pacific, 2013. **125**(925): p. 306-312.

14. Plummer, M., et al., *CODA: convergence diagnosis and output analysis for MCMC.* R News, 2006. **6**(1): p. 7-11.

15. Chang, K.C., et al., *Uncertainty Quantification Reveals the Importance of Data Variability and Experimental Design Considerations for in Silico Proarrhythmia Risk Assessment.* Frontiers in Physiology, 2017. **8**(917).

16. Li, Z., et al., *Assessment of an In Silico Mechanistic Model for Proarrhythmia Risk Prediction Under the CiPA Initiative.* Clinical Pharmacology & Therapeutics, 2019. **105**(2): p. 466-475.

17. Milnes, J.T., et al., *Investigating dynamic protocol-dependence of hERG potassium channel inhibition at 37°C: Cisapride versus dofetilide.* Journal of Pharmacological and Toxicological Methods, 2010. **61**(2): p. 178-191.

18. Arnu, H.T.O.M.D., *Covariance Matrix Adapting Evolutionary Strategy*. 2011, CRAN.

19. Li, Z., et al., *Improving the in silico assessment of proarrhythmia risk by combining hERG (human ether-à-go-go-related gene) channel–drug binding kinetics and multichannel pharmacology.* Circulation: Arrhythmia and Electrophysiology, 2017. **10**(2): p. e004628.

20. Soetaert, K., T. Petzoldt, and R.W. Setzer, *Solving Differential Equations in R: Package deSolve.* 2010, 2010. **33**(9): p. 25.

21. Li, Z., et al., *Improving the In Silico Assessment of Proarrhythmia Risk by Combining hERG (Human Ether-a-go-go-Related Gene) Channel-Drug Binding Kinetics and Multichannel Pharmacology.* Circ Arrhythm Electrophysiol, 2017. **10**(2): p. e004628.

22. Mistry, H.B., *Comprehensive In Vitro Proarrhythmic Assay Complexity Bias.* Clinical Pharmacology & Therapeutics, 2019. **105**(6): p. 1323-1324.

23. Sheng, J., et al., *Characterization of loperamide-mediated block of hERG channels at physiological temperature and its proarrhythmia propensity.* Journal of Pharmacological and Toxicological Methods, 2017. **88**: p. 109-122.

24. Studenik, C.R., Z. Zhou, and C.T. January, *Differences in action potential and early afterdepolarization properties in LQT2 and LQT3 models of long QT syndrome.* British Journal of Pharmacology, 2001. **132**(1): p. 85-92.

25. Zhou, Z., et al., *Properties of HERG Channels Stably Expressed in HEK 293 Cells Studied at Physiological Temperature.* Biophysical Journal, 1998. **74**(1): p. 230-241.
